# The relationship between tree size and tree water-use: is competition for water size-symmetric or size-asymmetric?

**DOI:** 10.1093/treephys/tpac018

**Published:** 2022-02-14

**Authors:** David I Forrester, Jean-Marc Limousin, Sebastian Pfautsch

**Affiliations:** Swiss Federal Institute of Forest, Snow and Landscape Research WSL, Zürcherstrasse 111, 8903 Birmensdorf, Switzerland; CSIRO Land and Water, GPO Box 1700, Canberra, ACT 2601, Australia; CEFE, Université de Montpellier, CNRS, EPHE, IRD, 1919 route de Mende, Montpellier Cedex 5 34293, France; Urban Studies, School of Social Science, Western Sydney University, Locked Bag 1797, Penrith, NSW 2751, Australia

**Keywords:** allometry, metabolic scaling theory, resource partitioning, sap flux density, SAPFLUXNET, sapwood area, transpiration

## Abstract

Relationships between tree size and water use indicate how soil water is partitioned between differently sized individuals, and hence competition for water. These relationships are rarely examined, let alone whether there is consistency in shape across populations. Competition for water among plants is often assumed to be size-symmetric, i.e., exponents (b_1_) of power functions (water use ∝ biomass^b1^) equal to 1, with all sizes using the same amount of water proportionally to their size. We tested the hypothesis that b_1_ actually varies greatly, and based on allometric theory, that b_1_ is only centered around 1 when size is quantified as basal area or sapwood area (not diameter). We also examined whether b_1_ varies spatially and temporally in relation to stand structure (height and density) and climate. Tree water use ∝ size^b1^ power functions were fitted for 80 species and 103 sites using the global SAPFLUXNET database. The b_1_ were centered around 1 when tree size was given as basal area or sapwood area, but not as diameter. The 95% confidence intervals of b_1_ included the theoretical predictions for the scaling of plant vascular networks. b_1_ changed through time within a given stand for the species with the longest time series, such that larger trees gained an advantage during warmer and wetter conditions. Spatial comparisons across the entire dataset showed that b_1_ correlated only weakly (*R*^2^ < 12%) with stand structure or climate, suggesting that inter-specific variability in b_1_ and hence the symmetry of competition for water may be largely related to inter-specific differences in tree architecture or physiology rather than to climate or stand structure. In conclusion, size-symmetric competition for water (b_1_ ≈ 1) may only be assumed when size is quantified as basal area or sapwood area, and when describing a general pattern across forest types and species. There is substantial deviation in b_1_ between individual stands and species.

## Introduction

**Figure 1.**
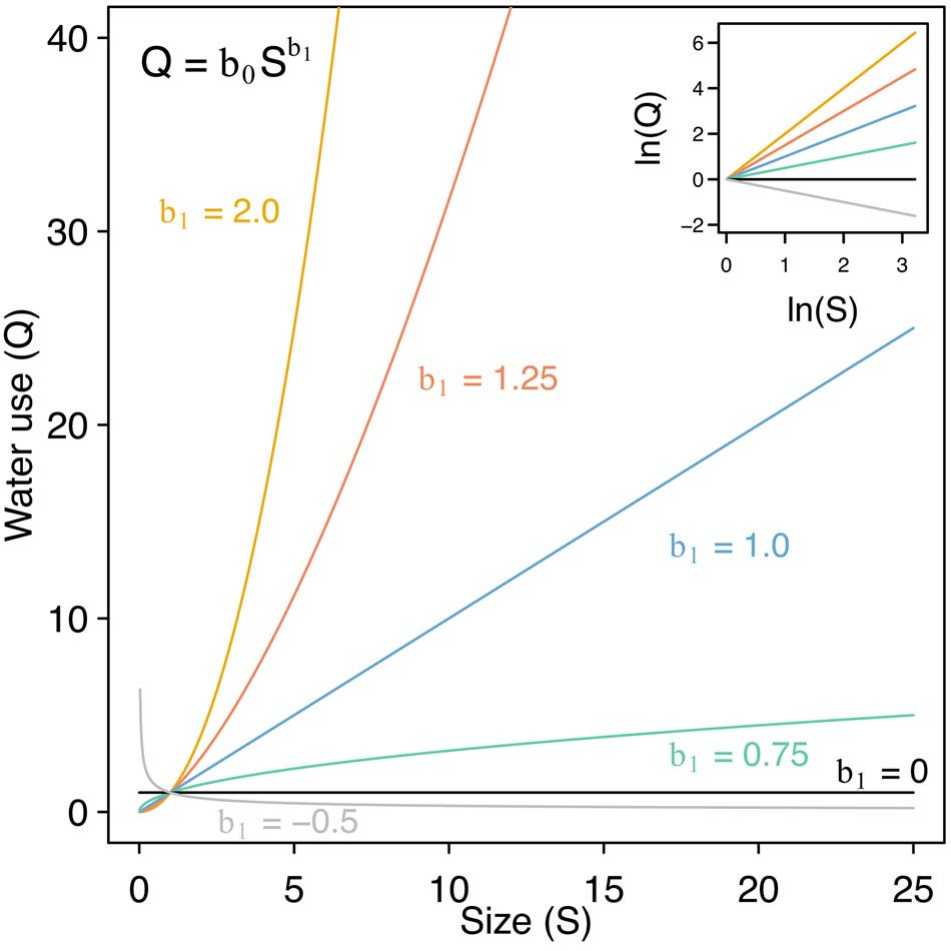
Contrasting shapes of size–water use relationships, described using a power function, for different values of exponent b_1_ (and b_0_ = 1). The inset shows the same lines after a natural logarithmic transformation. The equation implies that a 1% change in size (S) will result in a b_1_% change in water use (Q).

The scaling of processes with tree size provides a basis for examining relationships between form and function, and how they are constrained (West et al. 1999, Enquist et al. 2000, Savage et al. 2010, Lehnebach et al. 2018). These relationships also indicate how a stand’s resources are partitioned to different sized individuals within the stand, and are often considered when examining plant competition (Schwinning and Weiner 1998, Fernández-Tschieder and Binkley 2018, Forrester 2019). Relationships between tree size and function are also important when linking patterns observed at the individual level to those at the stand level, and therefore when upscaling from the individual to the stand (Hara 1993, Forrester 2019). However, despite this biological and ecological importance, few studies have examined relationships between tree size and tree water use, or whether there is consistency in the shape (e.g., b_1_ in Figure 1), of these relationships across tree populations (Meinzer et al. 2005).

Several functional forms have been used to scale water use with tree size, including power functions (West et al. 1999, Enquist et al. 2000, Savage et al. 2010), and sigmoidal functions (Meinzer et al. 2005). The former is based on a general theory of resource distribution through hierarchical branching networks, the metabolic scaling theory (West et al. 1999, Enquist et al. 2000), which was further developed by Savage et al. (2010). Savage et al. predicted that water use ∝ diameter^2^ and water use ∝ biomass^3/4^. This implies that a 1% increase in diameter will be associated with a 2% increase in water use, and a 1% increase in biomass will be associated with a ¾% increase in water use. The same theory suggests that sapwood area ∝ diameter^2^ and considering that basal area ∝ diameter^2^, it follows that water use ∝ sapwood area^1^, water use ∝ basal area^1^ and sapwood area ∝ basal area^1^. Meinzer et al. (2005) suggested that several assumptions of the West et al. (1999) hypothesis are not strictly true, and used data from eight stands to propose that sigmoid shapes may be more appropriate for size–water use relationships, at least for angiosperms.

When examining size–water use relationships it is useful to also examine the components of this relationship. Water use is often calculated as the product of sapwood area and sap flux density, which is the sap velocity per unit sapwood area (cm^3^ cm^−2^ h^−1^). According to the scaling theory presented by Savage et al. (2010), sap flux density ∝ diameter^0^, and it follows that sap flux density ∝ basal area^0^ and sap flux density ∝ sapwood area^0^. That is, the theory predicts that sap flux density does not change with increasing tree size.

A widely used implication of the shapes of the water-use relationships, e.g., as defined by b_1_ in Figure 1, relates to how different sized trees compete for water and therefore how a stand’s water resources are partitioned between trees. It is often assumed that competition for below-ground resources such as water is size-symmetric, such that all plant sizes obtain the same amount of a resource per unit size (i.e., b_1_ = 1 in Figure 1). This assumption appears to originate from early hypotheses about the symmetry and asymmetry of competition (Weiner 1990, Weiner et al. 1997, Schwinning and Weiner 1998), but these authors also explained that few data were available to test this hypothesis, especially in terms of measurements of the actual resources as opposed to using growth as a proxy for resource use. The size-symmetry of competition for soil water may therefore be an oversimplification, and several studies have indeed found non-linear diameter–water use relationships (Wullschleger et al. 1998, Enquist et al. 2000, Meinzer et al. 2005, Jung et al. 2011, Chen et al. 2012, Forrester 2015, von Allmen et al. 2015).

An associated hypothesis was that competition for light is size-asymmetric (Weiner 1990, Weiner et al. 1997, Schwinning and Weiner 1998), and this has also proven to be an oversimplification. That is, light competition depends on which light-related interactions are occurring within the canopy, and examples of size-symmetric light competition indicate that size-asymmetric light competition cannot always be assumed, even if more commonly observed than size-symmetric light competition (Forrester 2019). Furthermore, given that different size variables (e.g., diameter, height, sapwood area and biomass) are not linearly related (Enquist et al. 2000, Pretzsch et al. 2012), even if water use or light are linearly related to one size variable, they will automatically not be linearly related to others. Therefore, the size variable needs to be considered carefully (Weiner and Thomas 1992, Ex and Smith 2014, Looney et al. 2018, Forrester 2019). When examining water use, sapwood area might be a useful size variable given that it represents the cross-sectional area of the stem that conducts water. The b_1_ of the sapwood area to water use relationship is 1, as predicted using the scaling theory of Savage et al. (2010), which would be consistent with the hypothesis that competition for water is often size-symmetric but only when the size variable is sapwood area or basal area.

For a given size variable, b_1_ values can also vary between species and stands. For example, when the size variable is diameter, b_1_ has been found to range from ~1 to 3 (Wullschleger et al. 1998, Enquist et al. 2000, Meinzer et al. 2005, Kunert et al. 2010, Jung et al. 2011, Kunert et al. 2012, Forrester 2015, von Allmen et al. 2015). This could still be consistent with theoretical expectations of b_1_ ≈ 2 for tree diameter if it results from the random error around b_1_. Indeed, variability in water use per unit size (intercept b_0_, as opposed to b_1_ in Figure 1) may be expected given the differences within and between species in terms of the structure and anatomy of the water-conducting tissue, or the amount of water that can be stored in plant tissues (Meinzer et al. 2005). For a given tree diameter, angiosperms, which are vessel-bearing species, can transport more water than gymnosperms, which are tracheid-bearing (Hacke et al. 2005, Meinzer et al. 2005, Cernusak et al. 2008). However, it is not clear whether these differences would simply lead to higher water use per unit size (higher b_0_), or also to differences in the rate of increase in water use with size (changes in b_1_).

Within species variability in b_1_ may result when different sized trees respond differently to a given stand structure or environmental condition. For example taller trees and trees with larger crowns can intercept more precipitation and funnel it down their stems towards their roots, thereby increasing water availability for their roots and reducing the proportion of precipitation available to other trees (Crockford and Richardson 2000, Schume et al. 2004). On the other hand, smaller trees may benefit from cooler and moister within-canopy air conditions than larger trees whose crowns are exposed to drier, warmer air above the canopy (Liu and Muller 1993, Niinemets and Valladares 2004, Grote et al. 2016).

In this study, we used the SAPFLUXNET dataset, which contains tree water-use data from many species and forest types around the world (Poyatos et al. 2021), to test three hypotheses. The first hypothesis (i) was that basal area- or sapwood area–water use relationships have b_1_ ≈ 1 (i.e., size-symmetric), whereas diameter–water use relationships have b_1_ ≈ 2 (size-asymmetric). The second and third hypotheses were that regardless of the size variable, there will be broad variability around these means because (ii) angiosperms have on average higher b_1_ than gymnosperms, and because (iii) size–water use relationships vary with climate and stand structure (spatially and temporally), especially during periods of low rainfall or high vapor pressure deficit.

## Materials and methods

### Tree and sap flow data

**Figure 2.**
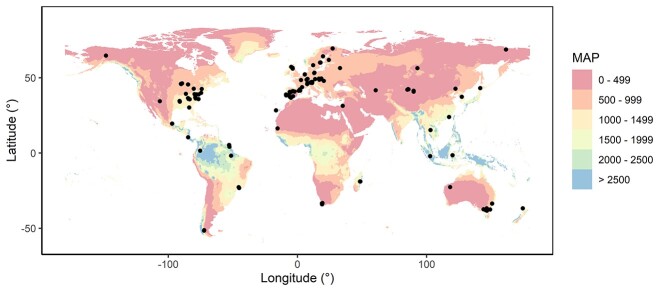
Locations of the sites from the SAPFLUXNET database included in this study, with a color gradient indicating the mean annual precipitation (MAP; mm) from WorldClim.

Whole tree water use, sap flux density, diameter, sapwood area and basal area data were obtained from the global SAPFLUXNET database (Poyatos et al. 2021). For each plot within the database, water use and sap flux density were representative of at least one growing season and included data for a minimum of 3 months. The recording frequencies ranged from 10 to 60 min (mean = 27 min), and the methods included heat dissipation methods, heat pulse methods and heat balance methods (Poyatos et al. 2021). Water use in the SAPFLUXNET dataset was calculated as sap flux density (cm^3^ cm^−2^ h^−1^) multiplied by sapwood area. Selection criterion for the present study was that water use data were available for at least five individual trees for a given species within a plot. This resulted in 2300 trees from 103 sites (141 plots), including 80 species (58 angiosperms and 22 gymnosperms; Figure 2,Figures S3–S15 available as Supplementary data at *Tree Physiology* Online). There were 215 species–plot combinations. A mean whole-tree water use (l day^−1^) and sap flux density (cm^3^ cm^−2^ h^−1^) value was calculated for each tree for its entire measurement period, or for each year in the case of analyses of temporal changes in size–water use relationships (Figures S3–S27 available as Supplementary data at *Tree Physiology* Online).

For each tree, the diameter at 1.3 m above ground was provided, and for 1769 trees, the sapwood area at 1.3 m was also available. We calculated the basal area of each tree, but did not consider the size variables height, volume or biomass, because these were measured for very few trees and additional errors would have been introduced by calculating them from equations not obtained from the sites. Size variables were measured once per tree.

The effects of temporal changes in climatic conditions on size–water use relationships were examined using three of the longest time series in the dataset. These included *Quercus ilex* from Puechabon, southern France (described in Limousin et al. 2009) measured from 2000 to 2015, and *Juniperus monosperma* and *Pinus edulis* from Sevilleta, New Mexico, USA (described in Pangle et al. 2012), measured from 2009 to 2015.

### Stand and climate data

The stand variables available to examine whether b_1_ was correlated with any stand characteristics, included stand basal area (m^2^ ha^−1^, *n* plots = 125), tree density (trees ha^−1^, *n* plots = 120), leaf area index (m^2^ m^−2^, *n* plots = 92), stand height (m, *n* plots = 135) and mean stand age (years, *n* plots = 130). The stand structures were also quantified in terms of the mean, median and maximum diameter (cm) of the sample trees for each species. Stand variables were measured once per site. Most plots were monospecific (*n* = 97), but 32 plots contained 2 species, and 11 plots contained 3–7 species.

Mean annual temperature (°C) and mean annual precipitation (mm) were available for all plots during the time when water use was measured. For the plots containing the three species with the longest time series, mean temperature (°C), precipitation (mm), incoming photosynthetic photon flux density (μmols m^−2^ s^−1^), vapor pressure deficit (kPa), soil water content (cm^3^ cm^−3^) and soil depth (cm) were available during the period when water use was measured.

### Statistical analyses

To test whether sigmoid (Eq. (1)) or power (Eq. (2)) functions best describe the size–water use relationships, both functional forms were fit for each species-stand combination and the Akaike Information Criterion (AIC) values were compared.

(1) \begin{equation*} Y=\frac{a}{1+{e}^{-b\left(X-c\right)}}, \end{equation*}

where *Y* is the response variable, *X* is the explanatory variable and *a*, *b* and *c* are fitted parameters.

(2) \begin{equation*} \ln (Y)=\ln\ {b}_0+{b}_1\ln (X) \end{equation*}

The sigmoid equations were fit as non-linear models and the power functions were fit as linear models with each variable ln-transformed.

Linear mixed models were used to examine whether the b_1_ of size–water use relationships differ between angiosperms and gymnosperms, between sap flow methodologies and between biomes. These were fit after combining all data. The site-stand-species identifier was used as a random variable.

Analysis of variance was used to test whether the mean b_1_ of size–water use power functions were significantly different from the b_1_ values predicted using the scaling theory. Analysis of variance was also used to test for differences in b_1_ due to sap flow methodologies and biome.

Linear regression was used to examine whether the b_1_ of diameter–water use relationships were correlated with stand and climate information. This was done after combining all data, and then separately for each of the three species with long time series.

All statistical analyses were performed using R 3.6.1 (R Development Core Team 2019). The mixed models were fit using the package *nlme* (Pinheiro et al. 2018).

## Results

Sigmoid functions often did not converge—only for 35 of 156 cases, compared with 136 for power functions (Table 1). When they did converge they only had lower AIC than power functions in 10 cases for all size variables. Visual inspection of plots where they did converge did not indicate that they were any better than power functions (Figures S3–S15 available as Supplementary data at *Tree Physiology* Online).

**Table 1:** Comparison of the number of significant power and sigmoid functions of water use as a function of size (diameter, basal area or sapwood area) fit to all plot–species combinations with at least 10 sample trees. The resulting functions are shown in Figures S3–S15 available as Supplementary data at *Tree Physiology* Online.

	Diameter	Basal area	Sapwood area	All
Plot–species combinations with *n* ≥ 10 trees sampled	52	52	51	155
Count of significant power functions	36	50	50	136
Count of significant sigmoid functions	22	5	8	35
Count of sigmoid functions with lower AIC than power functions	4	2	4	10

**Figure 3.**
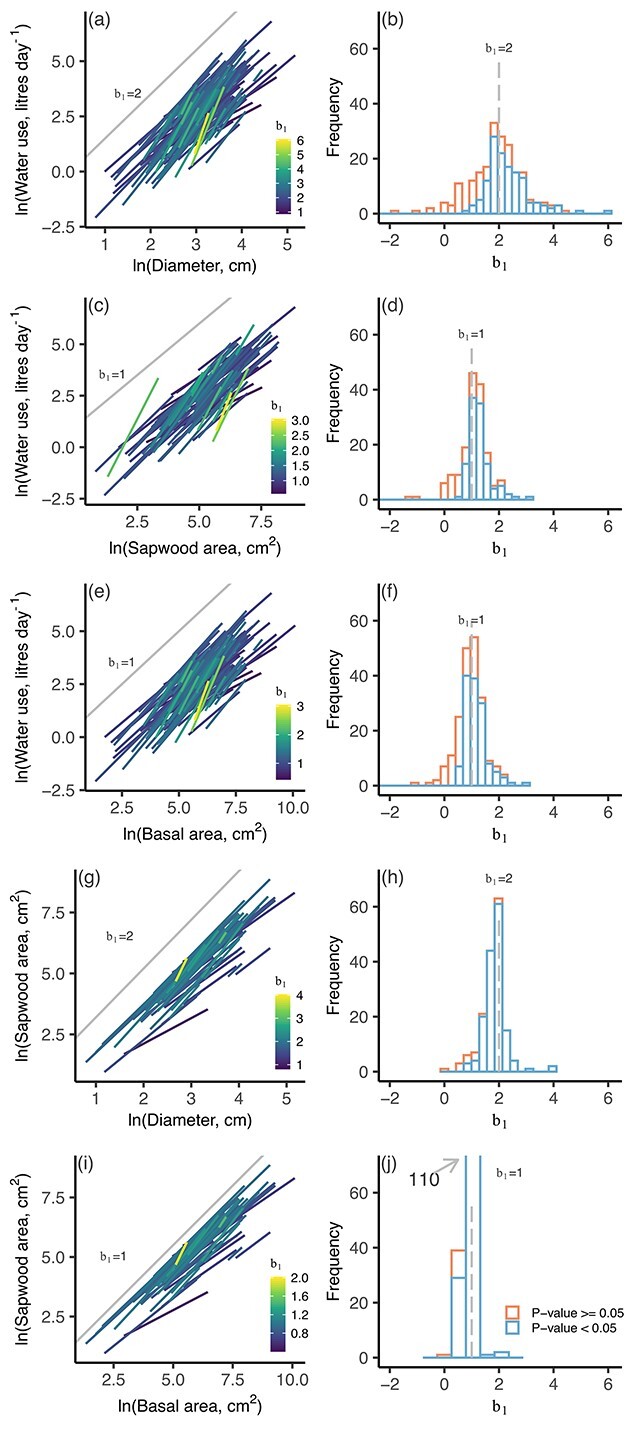
Linear relationships between ln-transformed water use and tree size variables for each species at each site (left column) and the frequency distribution of slopes of those relationships (b_1_; Eq. (1)) (right column). In the left column, lines are only shown for the relationships that were significant and where the slope was more than 0. Gray lines indicate the predicted b_1_ based on the scaling theory of Savage et al. (2010).

**Figure 4.**
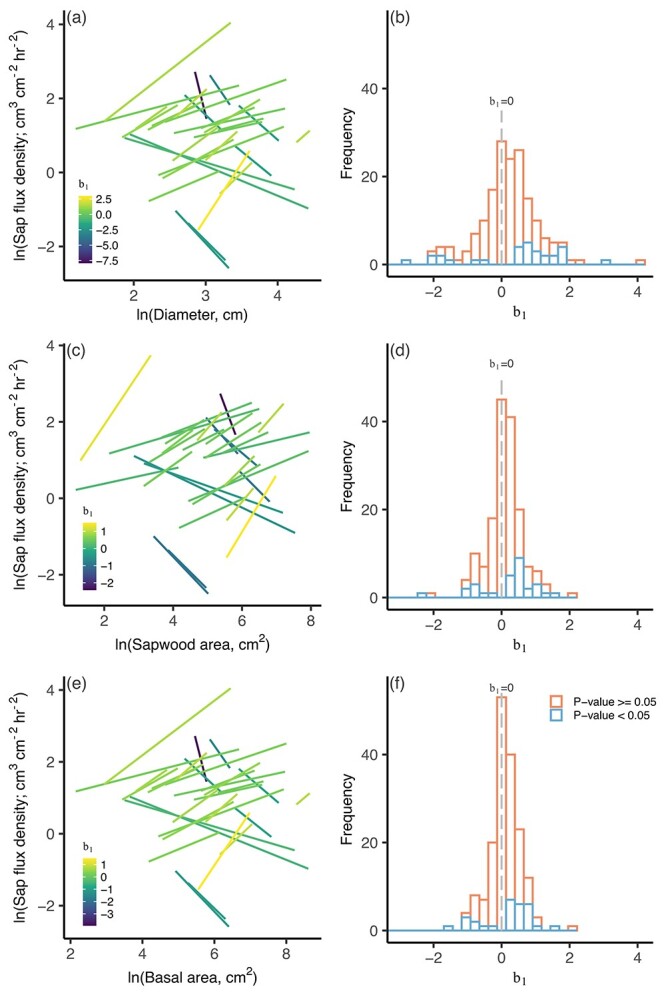
Linear relationships between ln-transformed sap flux density and tree size variables for each species at each site (left column) and the frequency distribution of slopes of those relationships (b_1_; Eq. (1)) (right column). In the left column, lines are only shown for the relationships that were significant. Gray lines indicate the predicted b_1_ based on the scaling theory of Savage et al. (2010).

The b_1_ of size–water use relationships were centered around the values predicted by the scaling theory of Savage et al. (2010) (Figure 3), as indicated by the 95% confidence intervals of the fitted b_1_ (Table 2). This was the case regardless of whether a single b_1_ was obtained for each species within each stand (Figure 3) or whether all species within a stand were combined (Figure S1 available as Supplementary data at *Tree Physiology* Online). Size–sap flux density relationships were often not significant (Figure 4). When they were significant, the 95% confidence interval included 0, which is the b_1_ predicted by the scaling theory of Savage et al. (2010) (Table 2).

**Table 2:** Comparison of measured b_1_ (based on SAPFLUXNET data) and the b_1_ predicted by the theory for plant network scaling (Savage et al. 2010). The mean, standard deviation and 95% confidence interval of the measured b_1_ are shown, as well as the sample sizes (*n*).

Independent variable	Dependent variable	Hypothesized b_1_	b_1_ mean	b_1_ standard deviation	*n*	95% confidence interval
b_1_ calculated for each species in each stand						
Diameter	Sapwood area	2	1.783	0.479	167	1.711 to 1.856
Basal area	Sapwood area	1	0.892	0.239	167	0.855 to 0.928
Diameter	Water use	2	1.937	1.158	206	1.779 to 2.095
Basal area	Water use	1	0.968	0.579	206	0.889 to 1.048
Sapwood area	Water use	1	1.064	0.681	167	0.961 to 1.167
Diameter	Sap flux density^1^	0	0.157	1.111	174	−0.008 to 0.322
Basal area	Sap flux density^1^	0	0.087	0.555	174	−0.004 to 0.161
Sapwood area	Sap flux density^1^	0	0.070	0.680	166	−0.034 to 0.173
b_1_ calculated for each stand (all species combined)						
Diameter	Sapwood area	2	1.718	0.546	118	1.62 to 1.817
Basal area	Sapwood area	1	0.859	0.273	118	0.81 to 0.908
Diameter	Water use	2	1.896	1.227	154	1.702 to 2.09
Basal area	Water use	1	0.948	0.614	154	0.851 to 1.045
Sapwood area	Water use	1	1.043	0.729	118	0.912 to 1.175
Diameter	Sap flux density^1^	0	0.163	1.176	123	−0.044 to 0.371
Basal area	Sap flux density^1^	0	0.082	0.588	123	−0.022 to 0.186
Sapwood area	Sap flux density^1^	0	0.051	0.728	117	−0.081 to 0.183

^1^Note that for > 82% of cases the sap flux density—size relationships were not significant, and while these are included in the *n*, they are not considered when calculating the other statistics.

**Figure 5.**
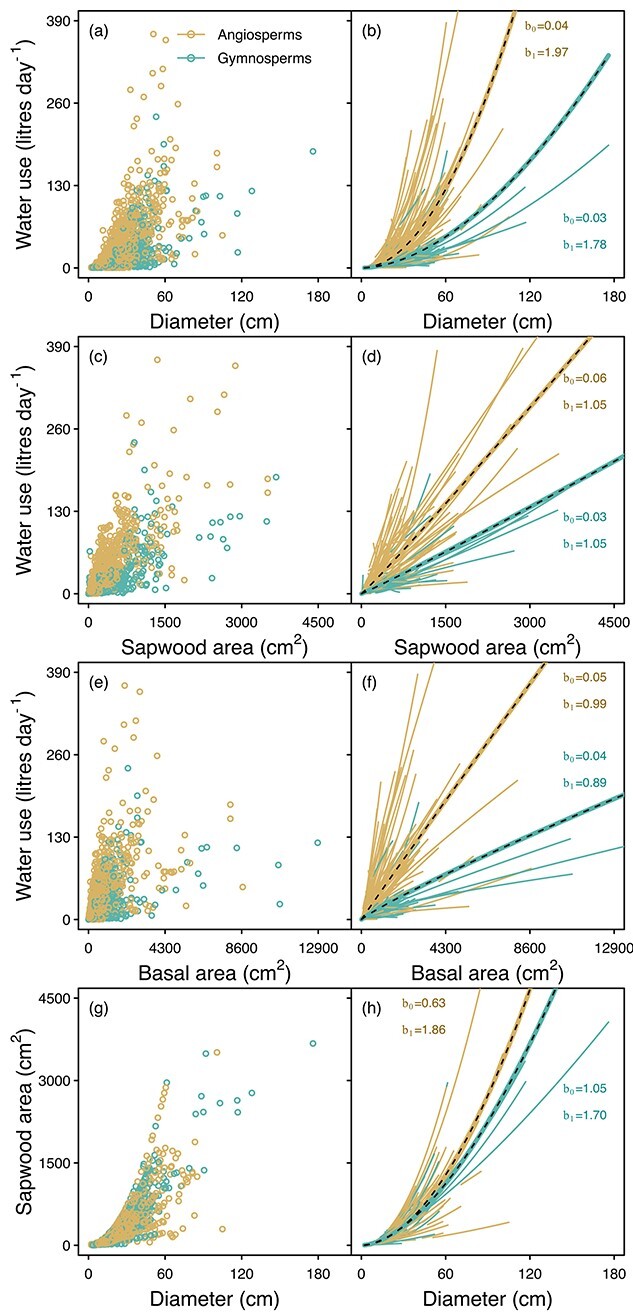
Differences between angiosperms and gymnosperms in terms of the relationships between sap flow and size variables. Individual-tree data points shown in the left column and lines fitted to each species in each stand (Eq. (2); Figure 1) are shown in the right column. The black dashed lines are the mean relationships for each group, and the b values associated with these lines are also indicated (additional statistics are provided in Table S1 available as Supplementary data at *Tree Physiology* Online).

Although the mean b_1_ of size–water use relationships were similar to the scaling theory predictions, there was a lot of variability around the means, with angiosperms having higher values than gymnosperms, on average, when the size variable was diameter or basal area (Figure 5 and Table S1 available as Supplementary data at *Tree Physiology* Online). There were no significant differences due to methodology or biome (Figure S2 available as Supplementary data at *Tree Physiology* Online).

**Figure 6.**
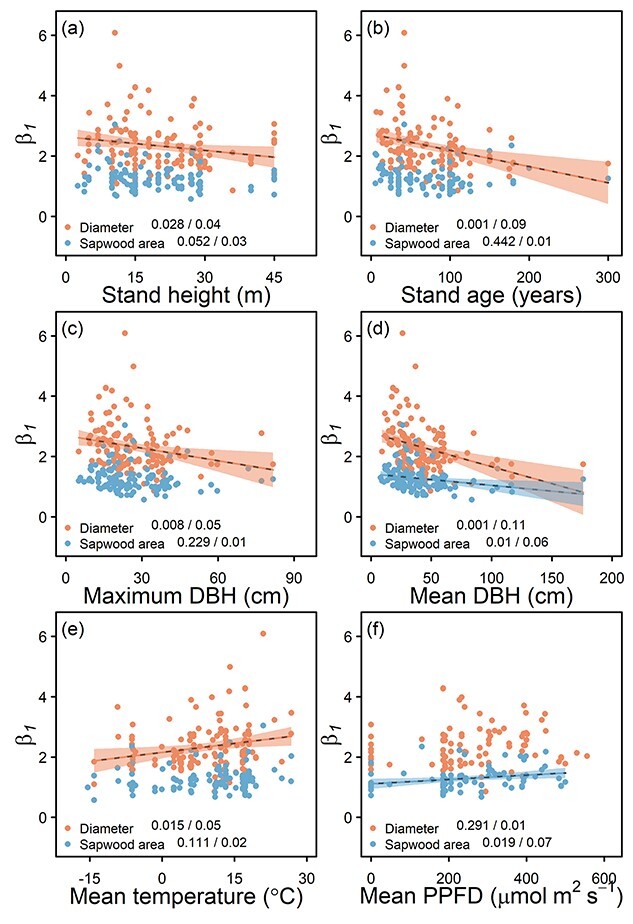
The effect of age and stand structural characteristics and climatic conditions on the b_1_ of size–water use relationships where size is quantified in terms of diameter or sapwood area. The *P*-value/adjusted *R*^2^ values are shown for each size variable and the black dotted lines are shown for relationships where *P* < 0.05. The shaded regions around the lines show 95% confidence intervals. PPFD is photosynthetic photon flux density.

The b_1_ of diameter-water use relationships were negatively correlated with stand height, stand age, mean diameter and maximum diameter, whereas they were positively correlated with mean temperature and photosynthetic photon flux density (Figure 6). However, these relationships were weak (*R*^2^ ≤ 0.11) although significant. There were no significant relationships with stand basal area, tree density, leaf area index or precipitation.

**Figure 7.**
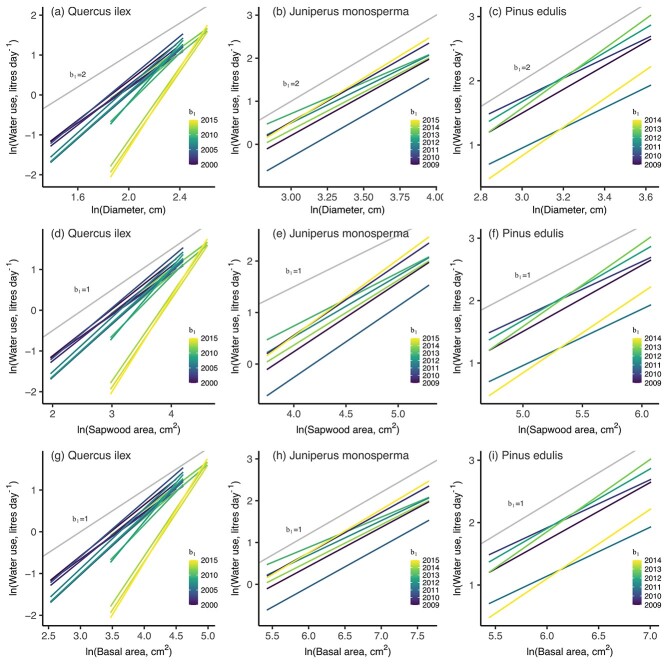
Linear relationships between ln-transformed water use and tree size variables for the three species that had been monitored for at least 10 years (calendar years shown in legends). Gray lines indicate the predicted b_1_ based on the scaling theory of Savage et al. (2010).

**Figure 8.**
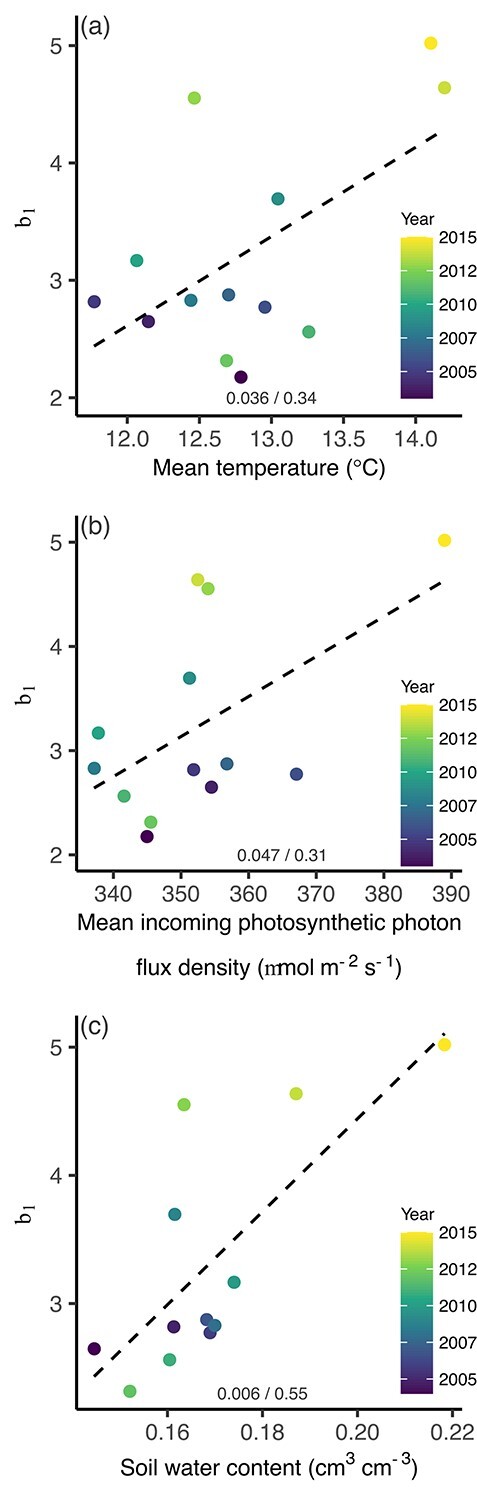
Changes in b_1_ in relation to temporal changes in mean temperature, photosynthetic photon flux density and soil water content at the site with the longest time series of water use data (*Quercus ilex* L. at Puechabon in southern France). The size variable was diameter. The *P*-value/adjusted *R*^2^ values are shown at the bottom for each of the fitted lines.

There was large temporal variability in b_1_ of diameter–water use relationships for the three species examined in the longest time series (Figure 7). For *Q. ilex*, the b_1_ were positively correlated with mean temperature, photon flux density and soil water content (Figure 8). However, no such correlations were significant for the other two species.

## Discussion

### Power functions versus sigmoidal functions

Size–water use relationships were usually described better using power functions than sigmoidal functions, regardless of whether the species was an angiosperm or gymnosperm or whether there were large trees in the sample that might have led to a sigmoidal shape. This contrasts with findings that sigmoidal shapes better describe diameter- or sapwood area–water use relationships for angiosperms (Meinzer et al. 2005), but it is consistent with the use of power functions for various scaling theories (West et al. 1999, Enquist et al. 2000, Savage et al. 2010).

### *Mean b_1_ and the scaling theory of*  Savage et al. *(2010)*

The b_1_ of size–water use relationships were centered around the values predicted by the scaling theory of Savage et al. (2010) regardless of whether the size variable was diameter, basal area or sapwood area. Interestingly, the b_1_ of diameter–sapwood area or basal area-sapwood area relationships were lower than expected based on the scaling theory. It is important to note that despite the relevance of the scaling theory to this study, it was not our objective to validate the theory. However, future studies that focus on its validation may need to carefully consider the accuracy of sapwood area data and the radial changes in sap flux density from outer to inner conducting sapwood (Čermák and Nadezhdina 1998, Ford et al. 2004, Lu et al. 2004, Poyatos et al. 2007, Forrester et al. 2012, Forrester 2015). Poyatos et al. (2021) noted sapwood area determination as a potential limitation of the SAPFLUXNET dataset, which does not contain information about the accuracy of sapwood data or the method used to estimate sapwood area.

### Angiosperms vs gymnosperms

For all size variables, there was a lot of variability around the mean b_1_ for water-use relationships. Angiosperms had significantly, albeit slightly, higher b_0_ and b_1_ than gymnosperms. Therefore, not only did angiosperms, on average, use more water per unit size, but the higher rate of increase in water use per unit size (higher b_1_) indicated that they compete slightly more asymmetrically for water than gymnosperms. The higher water use (b_0_) for angiosperms may be associated with their greater root to shoot ratios (Reich et al. 2014, Qi et al. 2019), because they use vessels rather than tracheids to transport water (Hacke et al. 2005, Cernusak et al. 2008), and because the ratios of sapwood area to leaf area (Huber values) were much lower for angiosperms (3.9 cm^2^ m^−2^) than gymnosperms (15.1 cm^2^ m^−2^) in our dataset. The slightly higher asymmetry in water use by angiosperms suggests that plant interactions that favor large trees, in terms of water uptake, may be more common or more important for angiosperms. For example, if angiosperms have wider crowns (e.g., Forrester et al. 2017), they might be more likely to overtop shorter trees, which could be advantageous if their crowns intercept water and funnel it down to their roots, and away from the roots of shorter trees. However, we know of no studies that have examined this by measuring interception and water use of co-occurring angiosperms and gymnosperms.

### Stand structure and climatic conditions

The b_1_ were negatively correlated (i.e., large trees had less advantage) with increasing stand age, stand height and mean or maximum diameter. This suggests that competition for water becomes less asymmetric as the maximum size of trees within a population increases, e.g., in terms of height or diameter, or due to age. This may partly result if there are non-linear increases in hydraulic limitations with tree height (Ryan et al. 2006). Nevertheless, given that these were very weak correlations, and other stand structural characteristics (e.g., stand basal area, tree density and leaf area index) or climate were not correlated with b_1_, inter-specific differences in tree architecture or physiology may be more influential than the competitive environment.

### Temporal changes

The long time series enabled an examination of whether size–water use relationships vary through time. In all three populations, b_1_ varied between years, and for *Q. ilex*, b_1_ was positively correlated with temperature, photosynthetic photon flux density and soil water content. That is, competition for water became more asymmetric, thereby favoring larger *Q. ilex* individuals as environmental conditions favoring tree water use improved (temperature, light and soil water content). It is well known that larger trees may lose part of their advantage during harsher periods due to their greater hydraulic limitations, and because they experience greater evaporative demands higher up in the canopy when irradiance, temperature and vapour pressure deficit are higher (Liu and Muller 1993, Niinemets and Valladares 2004, Ryan et al. 2006, Niinemets 2010, Bennett et al. 2015, Grote et al. 2016). However, hydraulic limitations may not have caused the pattern observed in the *Q. ilex* trees, which were in an even-aged forest on a rocky site and only ~4–6 m tall. Instead, larger trees may have been growing in more favorable soil conditions, allowing them to achieve larger sizes at any given age by having access to more soil water and probably higher ratios of leaf area to sapwood area (Carrière et al. 2020).

### Is competition for water size-symmetric?

Although it is often assumed that competition for water is size-symmetric, this is only plausible when size is quantified as basal area or sapwood area. Furthermore, it is only plausible when considered as a general pattern across many forest types and species, and not when considering specific forests or species or points in time. That is, although the mean b_1_ was close to 1, most stands or species had b_1_ smaller or larger than 1. This reflects the fact that there are many different water-related interactions that favor different tree sizes (e.g., Forrester 2019), and the relative importance of these interactions is likely to vary between species, and with spatial and temporal changes in climatic conditions and stand structures.

The shape of size-growth relationships is often used to imply whether competition is more for soil resources or light. This is based on assumptions that competition for soil resources is size-symmetric, whereas competition for light is size-asymmetric (Weiner 1990, Weiner et al. 1997, Schwinning and Weiner 1998). However, size–water use relationships were often not size-symmetric (b_1_ ≠ 1; Figure 2), and size–light absorption relationships are often not size-asymmetric (Forrester 2019). Therefore, these assumptions are unlikely to be appropriate, especially for individual species, sites and ages. It is also unrealistic given that growth is not only a function of resource uptake but also depends on resource use efficiency (and hence climate, soils and biotic stressors) (Monteith 1977, Binkley et al. 2004). Therefore, even if size–water use relationships have b_1_ = 1, size–growth relationships may be strongly asymmetric (b_1_ > 1 or b_1_ < 1) due to size–resource use efficiency relationships with b_1_ > 1 or b_1_ < 1.

### Aggregated, not single tree analyses

It is important to note that all of the size–water use relationships in this study are aggregated analyses where all trees of a given species were used to fit a relationship at a single point in time. This contrasts with longitudinal analyses where the water use of a single tree is examined as its size increases through time. Although the aggregated relationships typically indicated continuously increasing water use with tree size (Figures S3–S15 available as Supplementary data at *Tree Physiology* Online), longitudinal relationships may sometimes show declines in water use as trees become larger, as found for relationships between tree biomass and biomass growth (Sheil et al. 2017, Forrester 2021). We did not have long enough time series for individual trees to further test this.

## Conclusions

The b_1_ of size–water use relationships as well as those of size–sap flux density relationships were centered around the values predicted by the scaling theory (Savage et al. 2010), regardless of whether the size variable was diameter, basal area or sapwood area.

There was considerable variability around the mean b_1_ of size–water use relationships. A very small amount of this variability was due to stand structure and temperature such that b_1_ values were slightly higher (i.e., more asymmetric) for shorter stands, younger stands and warmer sites, but there were no effects of stand basal area, tree density, leaf area index or precipitation. This suggests that the inter-specific variability in b_1_ and hence potentially the symmetry of competition for water, is related more to inter-specific differences in tree architecture or physiology than to the competitive environment. The b_1_ changed within a given stand through time, such that larger trees gained an advantage during years with more favorable climatic conditions.

Although it is often assumed that competition for water is size-symmetric, this is only plausible when size is quantified as basal area or sapwood area, and when describing a general pattern across many forest types and species. It is not a reliable assumption when considering specific forests or species or points in time. That is, while the mean b_1_ was close to 1, most stands or species had b_1_ smaller or larger than 1. This variability is expected given the wide range in tree physiological and allometric characteristics and hence in the potential water-related interactions between plants.

## Data availability statement

The data are available as part of the SAPFLUXNET database (Poyatos et al. 2021).

## Authors’ contributions

D.I.F. designed the study with input from J.-M.L., and S.P., D.I.F. performed the analyses and wrote the first draft of the manuscript. All authors commented critically consequent versions of the manuscript.

## Supplementary Material

Supp_Information_revised_tpac018
