# Supplementary material for: The relationship between tree size and tree water-use: is competition for water size-symmetric or size-asymmetric?
Source: Tree Physiol. 2022 Feb 14;42(10):1916–27. doi: 10.1093/treephys/tpac018 (PMC9838098; doi:10.1093/treephys/tpac018)
Supplement: Supp_Information_revised_tpac018 [file supp_information_revised_tpac018.pdf]

Table S1. Parameter estimates and their standard errors for models shown in Figure 4. The “G” indicates the change in the intercept (ln(b_0)) or slope (b_1) for the gymnosperms; the angiosperms were used as the base line in the models.

ln(diameter) - ln(water use); sample size = 2061			
Parameter	Estimate	se	P-values (ANOVA)
ln(b_0)	-3.256	0.133	<0.001
b_1	1.973	0.036	<0.001
ln(b_0) G	-0.157	0.219	<0.001
b_1 G	-0.188	0.058	0.001
**ln(sapwood area) - ln(water use); sample size = 1772**			
Parameter	Estimate	se	P-values (ANOVA)
ln(b_0)	-2.697	0.111	<0.001
b_1	1.047	0.016	<0.001
ln(b_0) G	-0.767	0.121	<0.001
b_1 G	ns		
**ln(basal area) - ln(water use); sample size = 2061**			
Parameter	Estimate	se	P-values (ANOVA)
ln(b_0)	-3.017	0.129	<0.001
b_1	0.986	0.018	<0.001
ln(b_0) G	-0.18	0.213	<0.001
b_1 G	-0.094	0.029	0.001
**ln(diameter) - ln(sapwood area); sample size = 1772**			
Parameter	Estimate	se	P-values (ANOVA)
ln(b_0)	-0.461	0.066	<0.001
b_1	1.86	0.013	<0.001
ln(b_0) G	0.506	0.109	0.871
b_1 G	-0.156	0.021	<0.001
**ln(basal area) - ln(sapwood area); sample size = 1773**			
Parameter	Estimate	se	P-values (ANOVA)
ln(b_0)	-0.236	0.065	<0.001
b_1	0.93	0.007	<0.001
ln(b_0) G	0.487	0.108	0.871
b_1 G	-0.078	0.011	<0.001

Figure S1. Linear relationships between ln-transformed water use and tree size variables for each stand (left column) and the frequency distribution of slopes of those relationships (b_1; Equation 1) (right column). In the left column, lines are only shown for the relationships that were significant and where the slope was more than 0. Note that this figure contains fewer lines than Figure 3 because it shows lines fitted for each stand, as opposed to each individual species. Grey lines indicate the predicted b_1 based on the metabolic scaling theory.

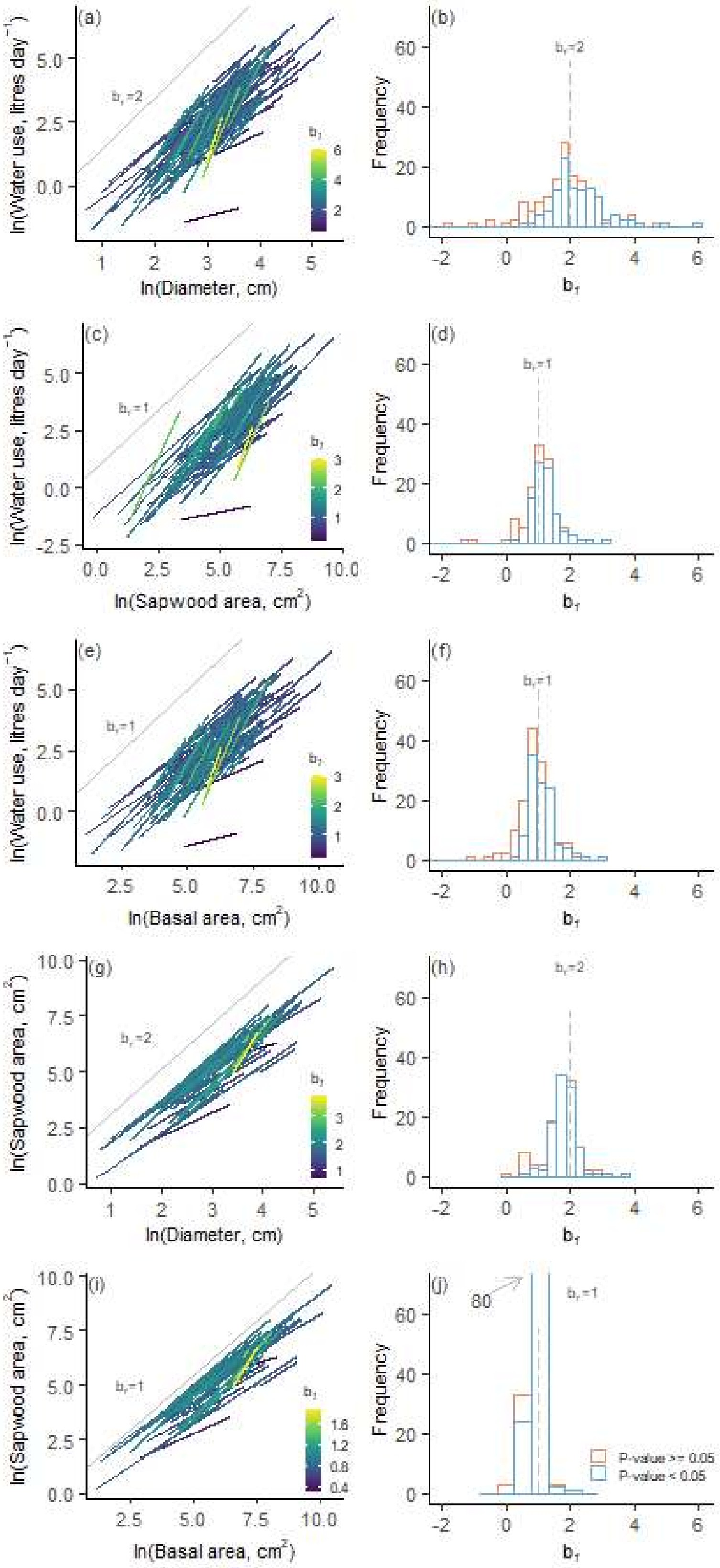

Figure S2. Frequency distributions and box plots for b_1 (Equation 1) of relationships between diameter and water use for different methodologies (a) and different biomes (b). Frequencies are based only on b_1 for significant relationships. There were no significant differences between methods or biomes. CHP = compensation heat pulse; HD = constant heat dissipation; HFD = heat deformation; HPTM = T-max; HR = heat ratio; TSHB = trunk sector heat balance; for detailed descriptions of methods see Poyatos *et al.* (2021).

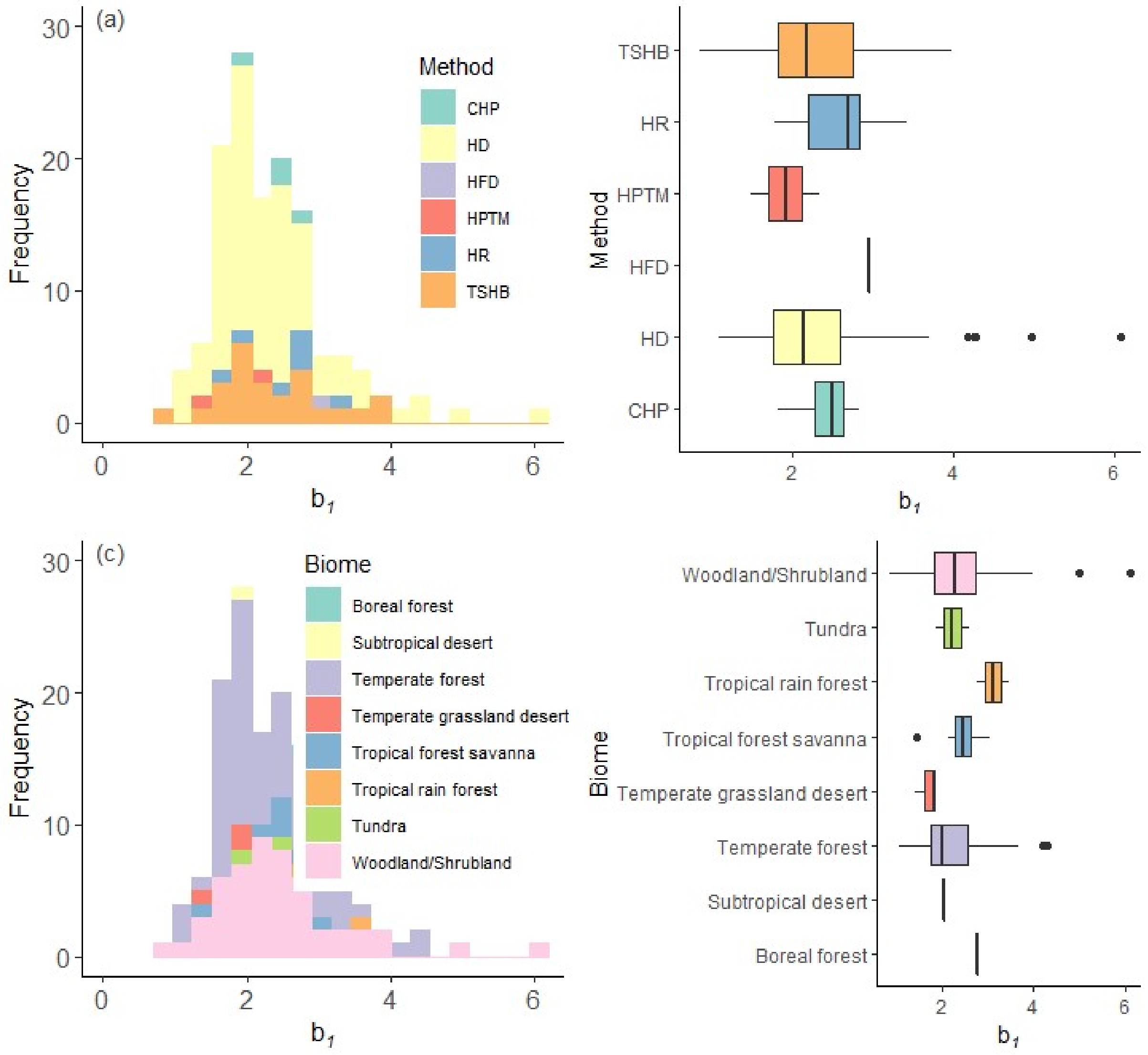

Figure S3. Diameter – water use relationships for individual species (figure 1 of 7). When significant, power functions are shown as black lines and sigmoidal functions are shown as red lines.

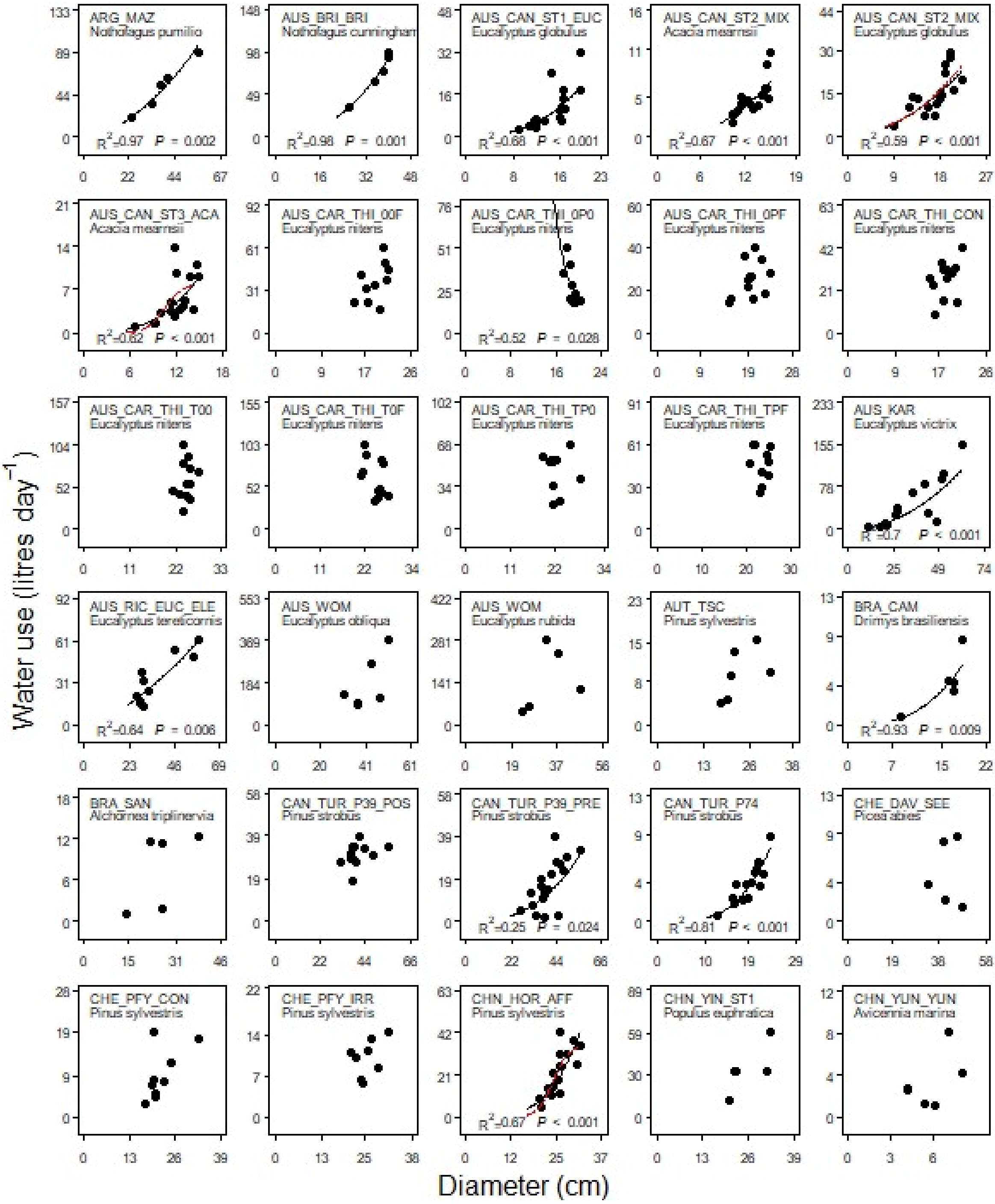

Figure S4. Diameter – water use relationships for individual species (figure 2 of 7). When significant, power functions are shown as black lines.

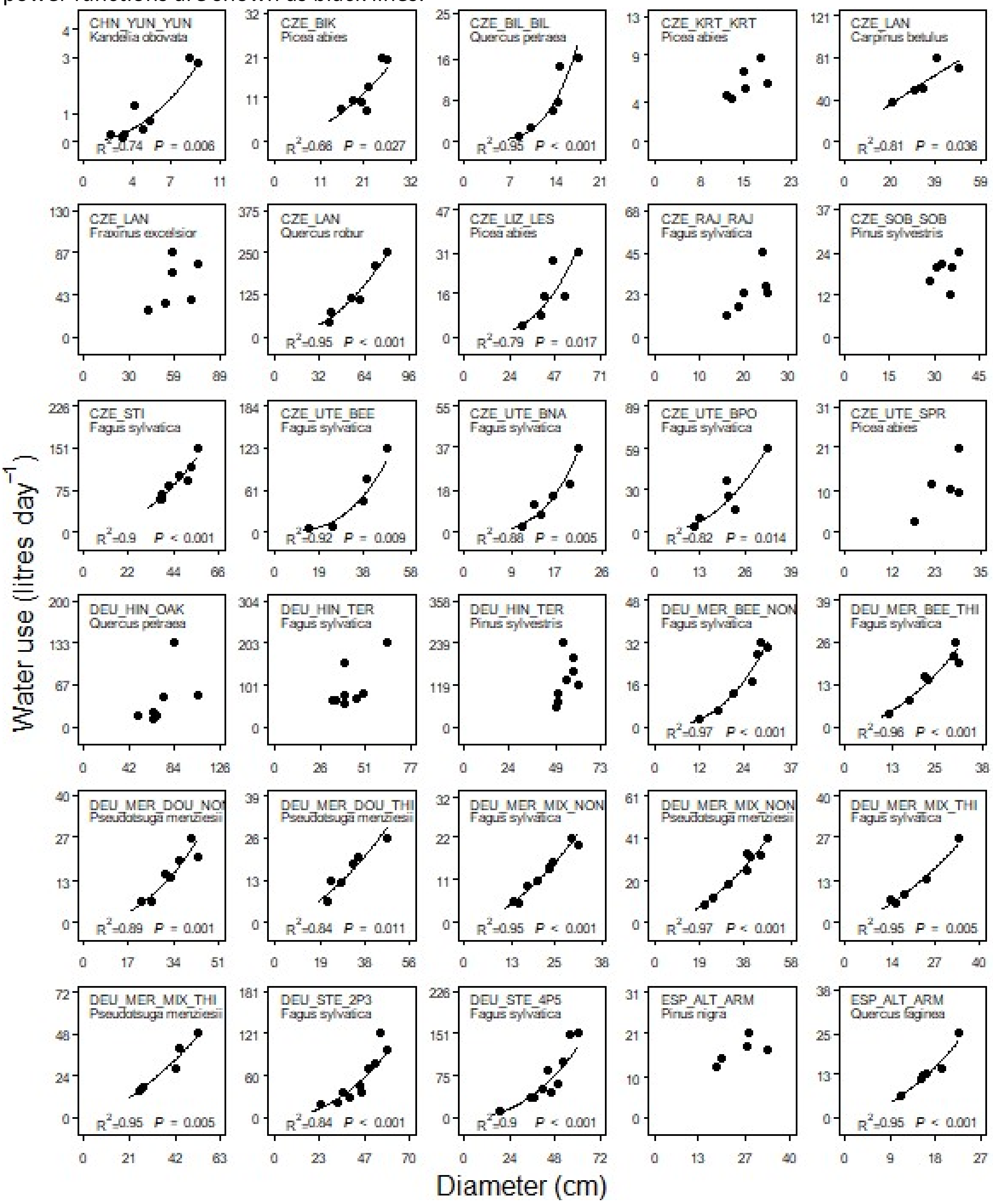

Figure S5. Diameter – water use relationships for individual species (figure 3 of 7). When significant, power functions are shown as black lines and sigmoidal functions are shown as red lines.

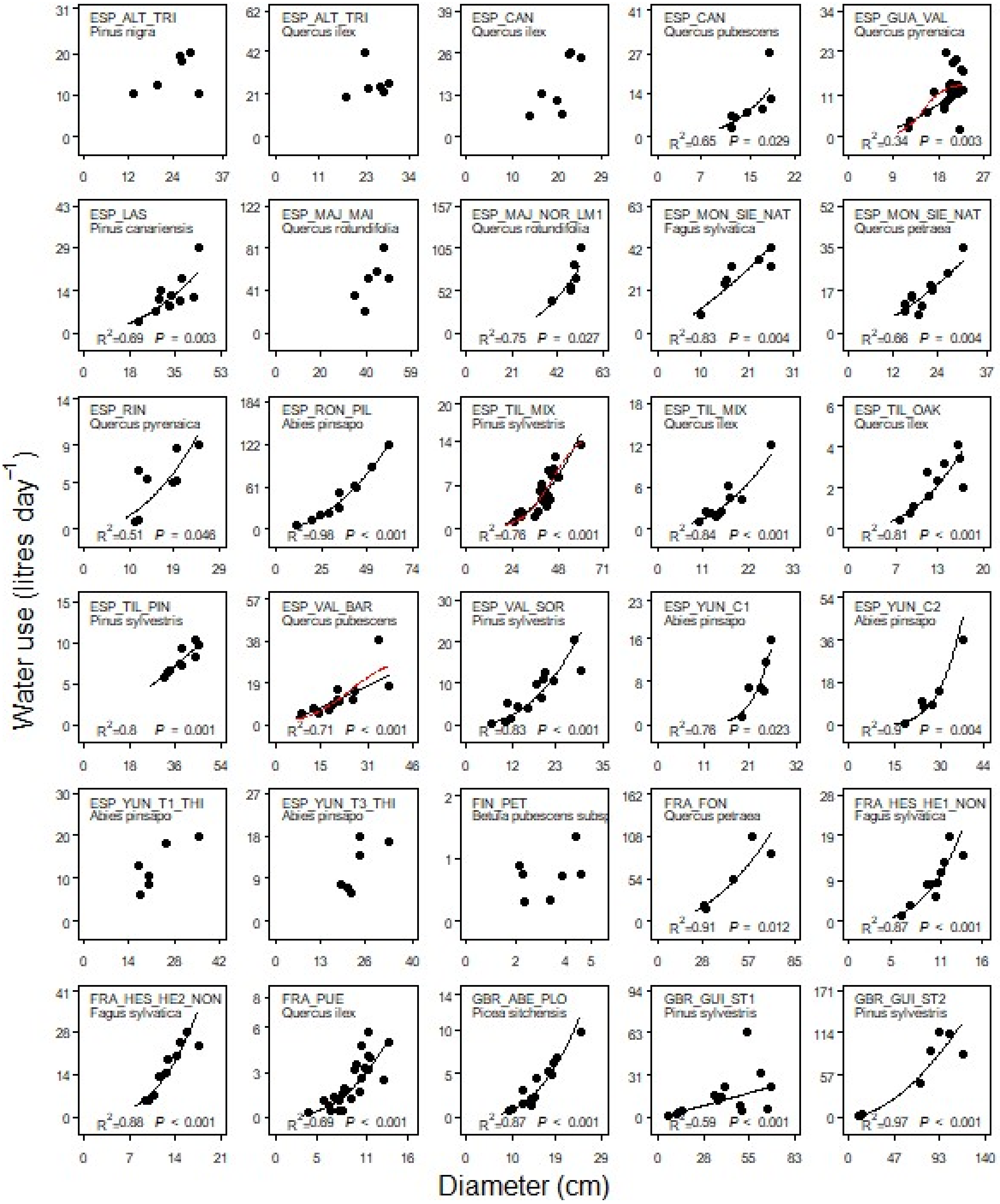

Figure S6. Diameter – water use relationships for individual species (figure 4 of 7). When significant, power functions are shown as black lines and sigmoidal functions are shown as red lines.

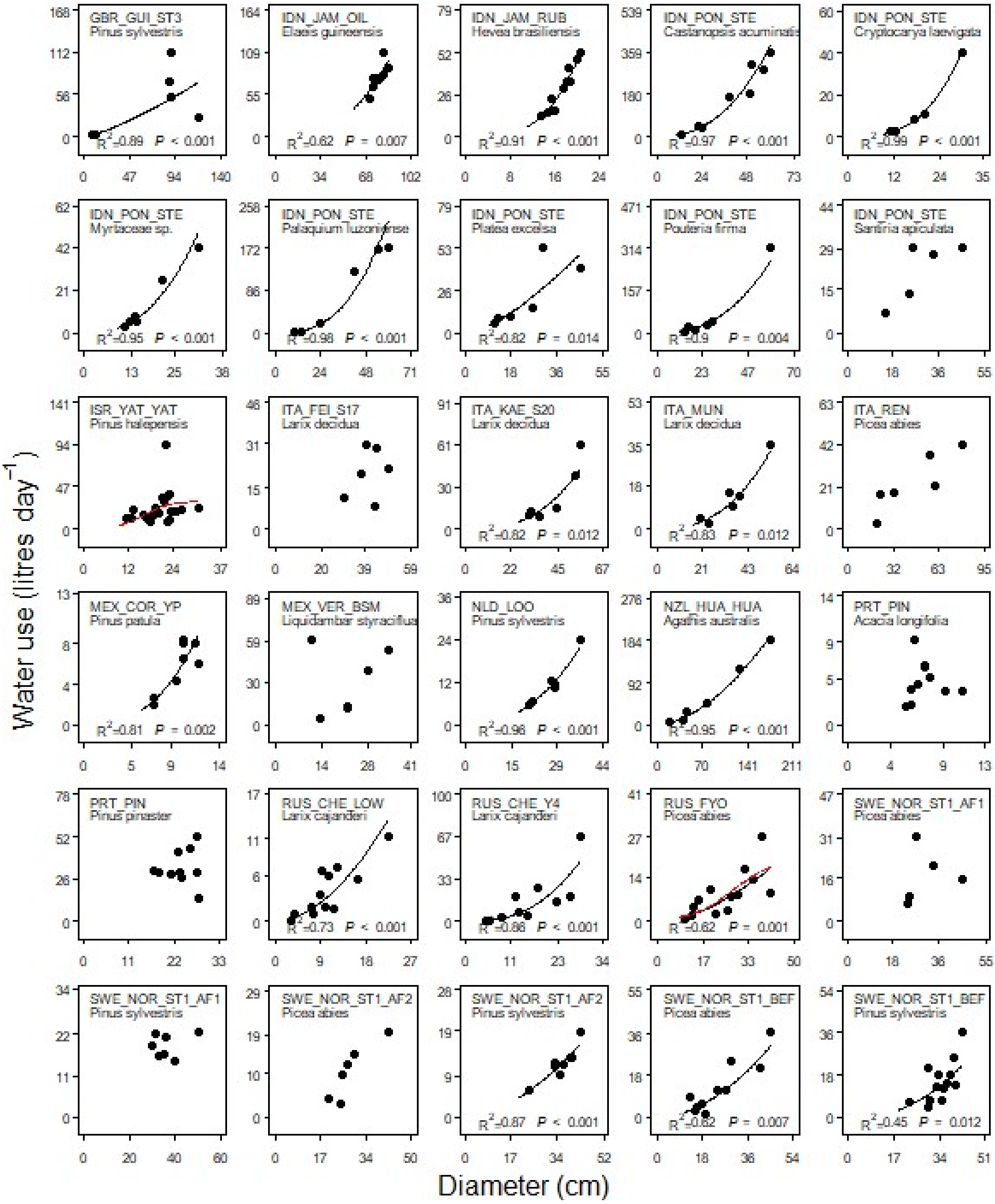

Figure S7. Diameter – water use relationships for individual species (figure 5 of 7). When significant, power functions are shown as black lines and sigmoidal functions are shown as red lines.

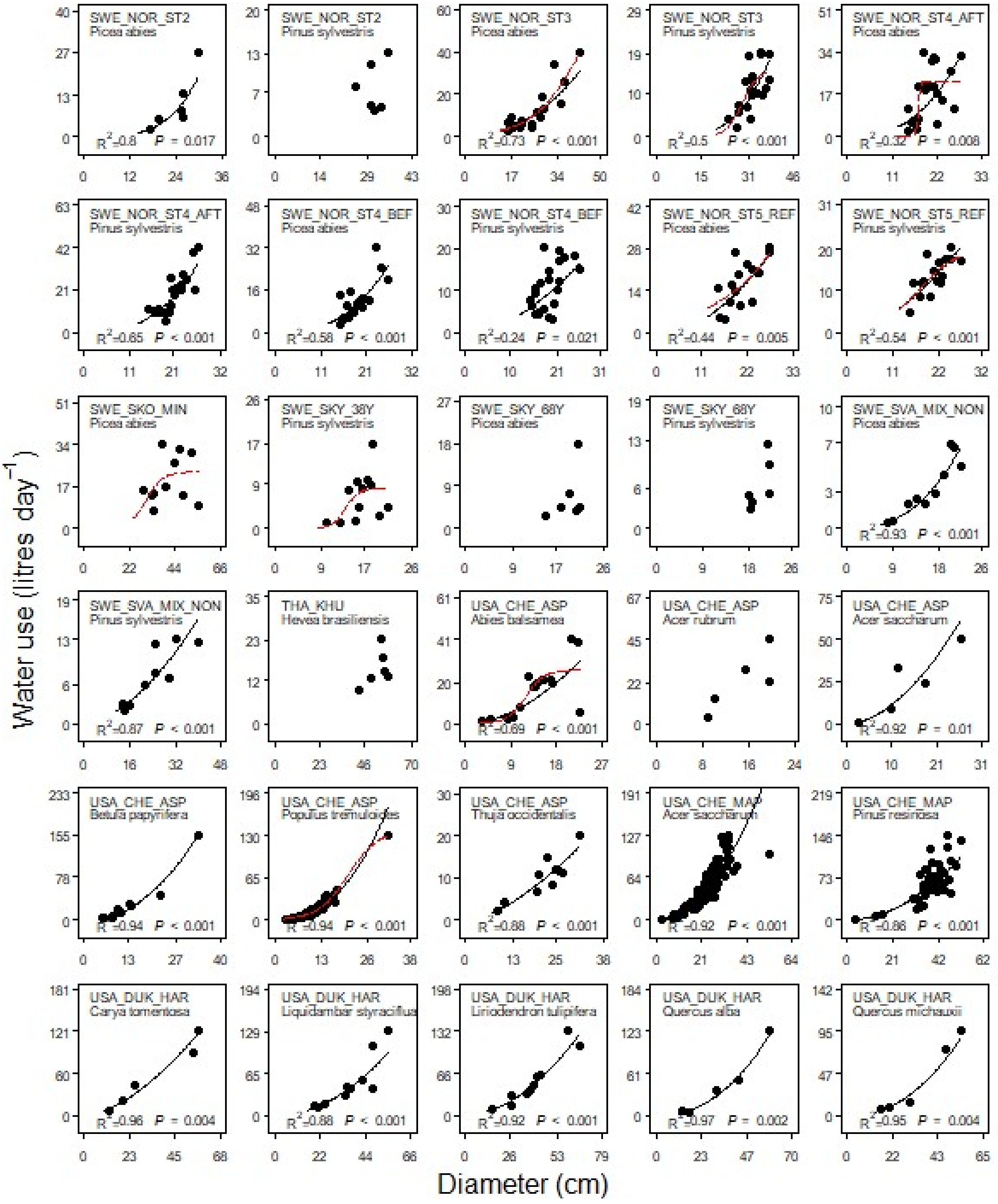

Figure S8. Diameter – water use relationships for individual species (figure 6 of 7). When significant, power functions are shown as black lines and sigmoidal functions are shown as red lines.

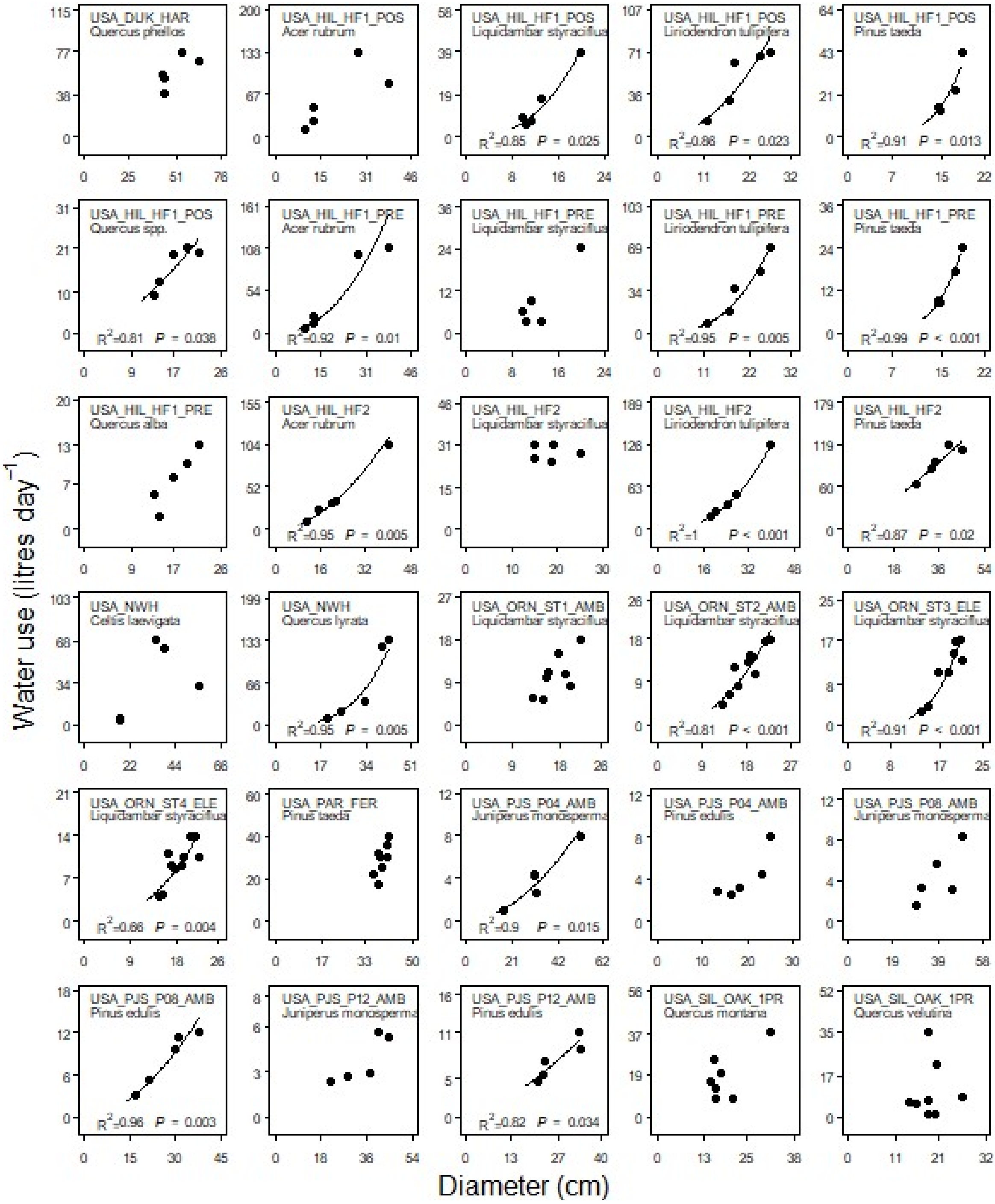

Figure S9. Diameter – water use relationships for individual species (figure 7 of 7). When significant, power functions are shown as black lines and sigmoidal functions are shown as red lines.

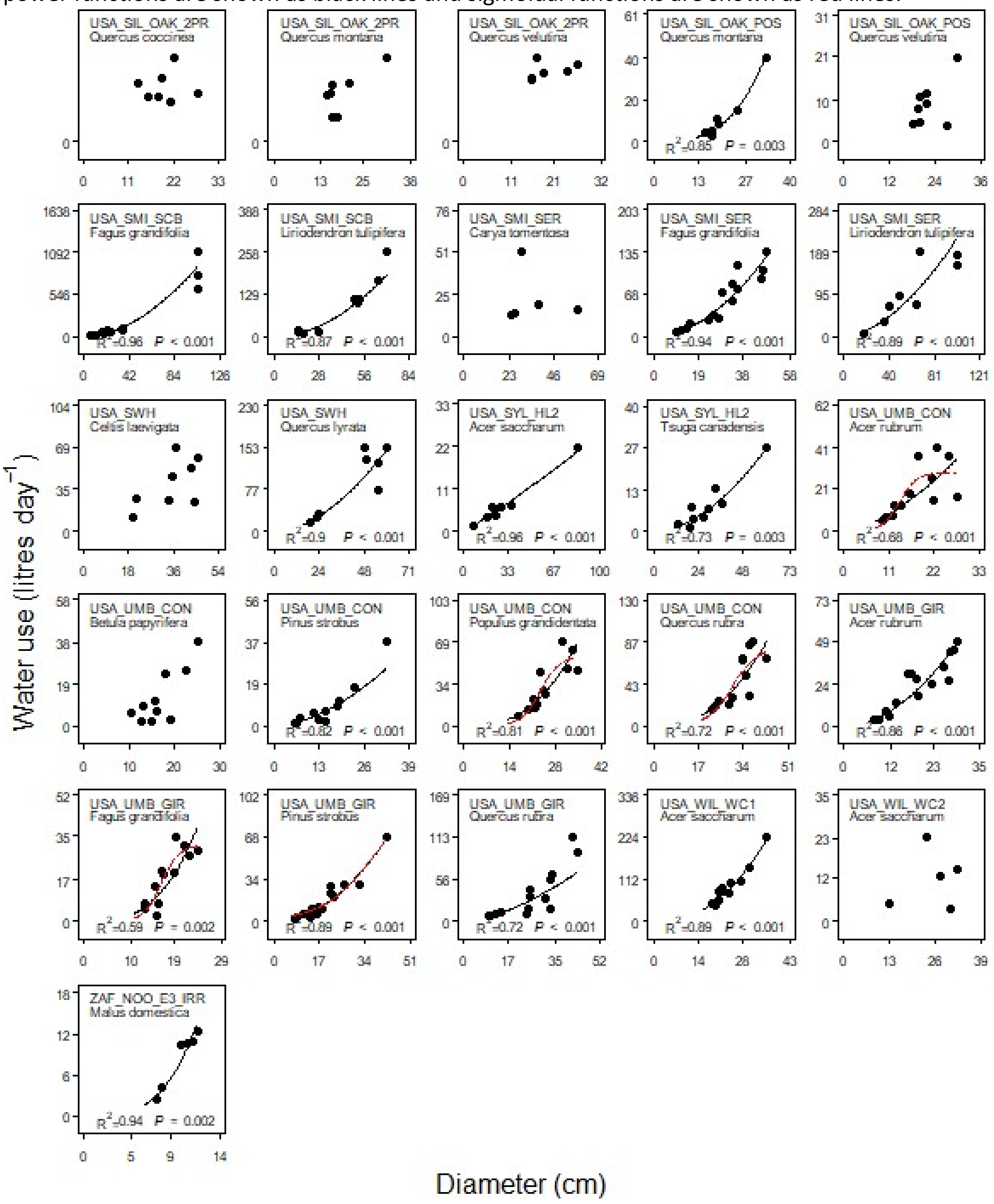

Figure S10. Sapwood area – water use relationships for individual species (figure 1 of 6). When significant, power functions are shown as black lines and sigmoidal functions are shown as red lines.

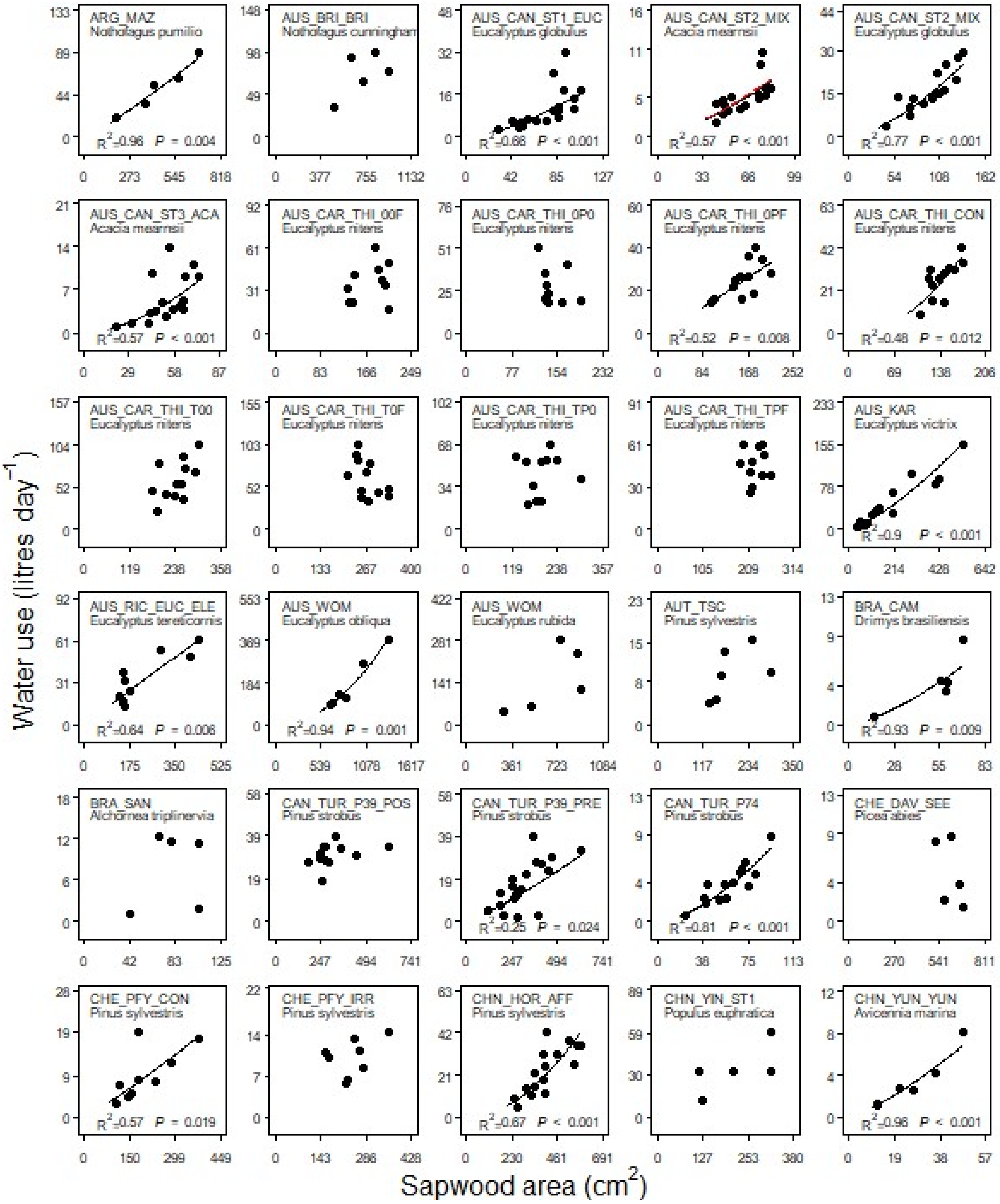

Figure S11. Sapwood area – water use relationships for individual species (figure 2 of 6). When significant, power functions are shown as black lines.

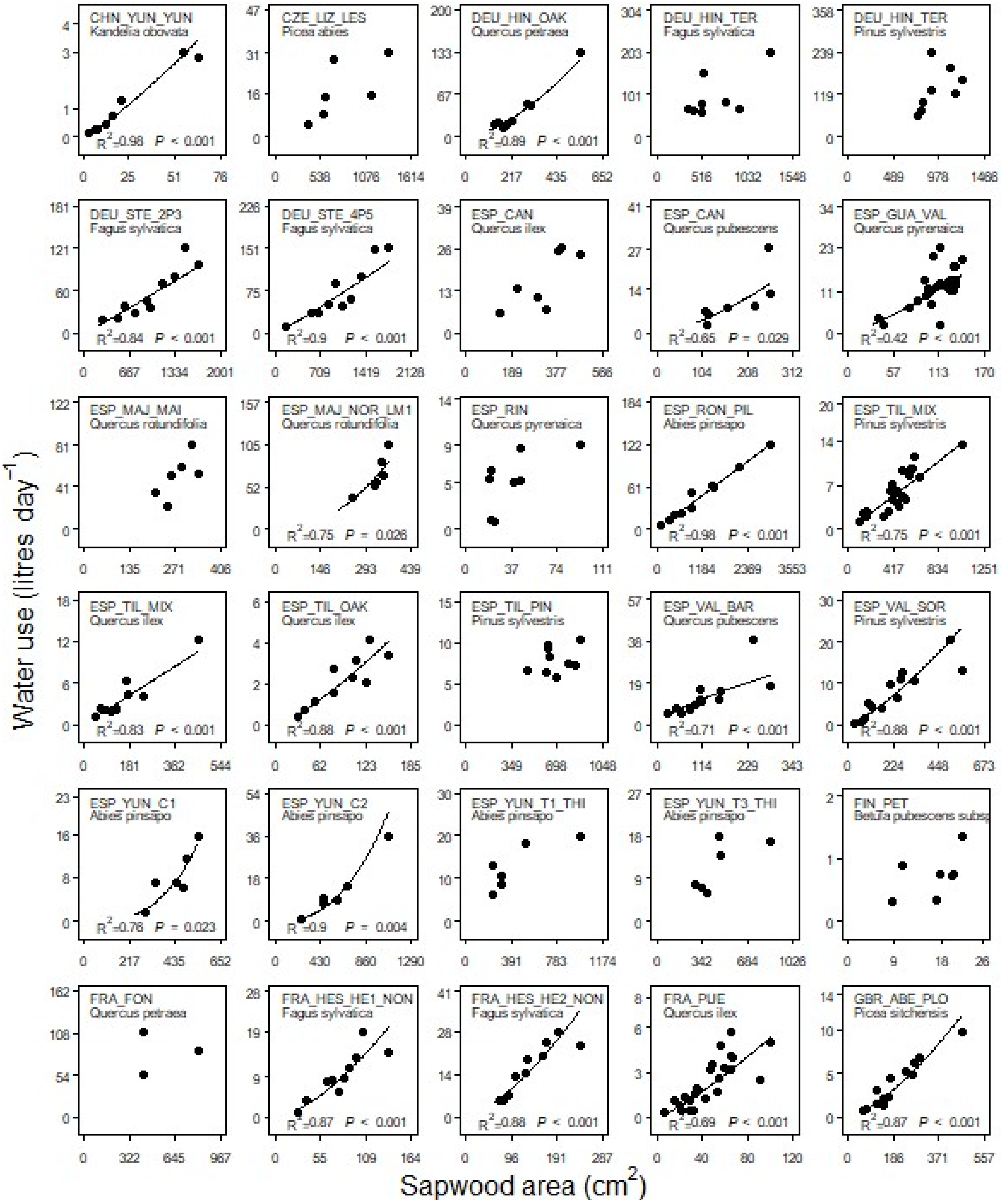

Figure S12. Sapwood area – water use relationships for individual species (figure 3 of 6). When significant, power functions are shown as black lines and sigmoidal functions are shown as red lines.

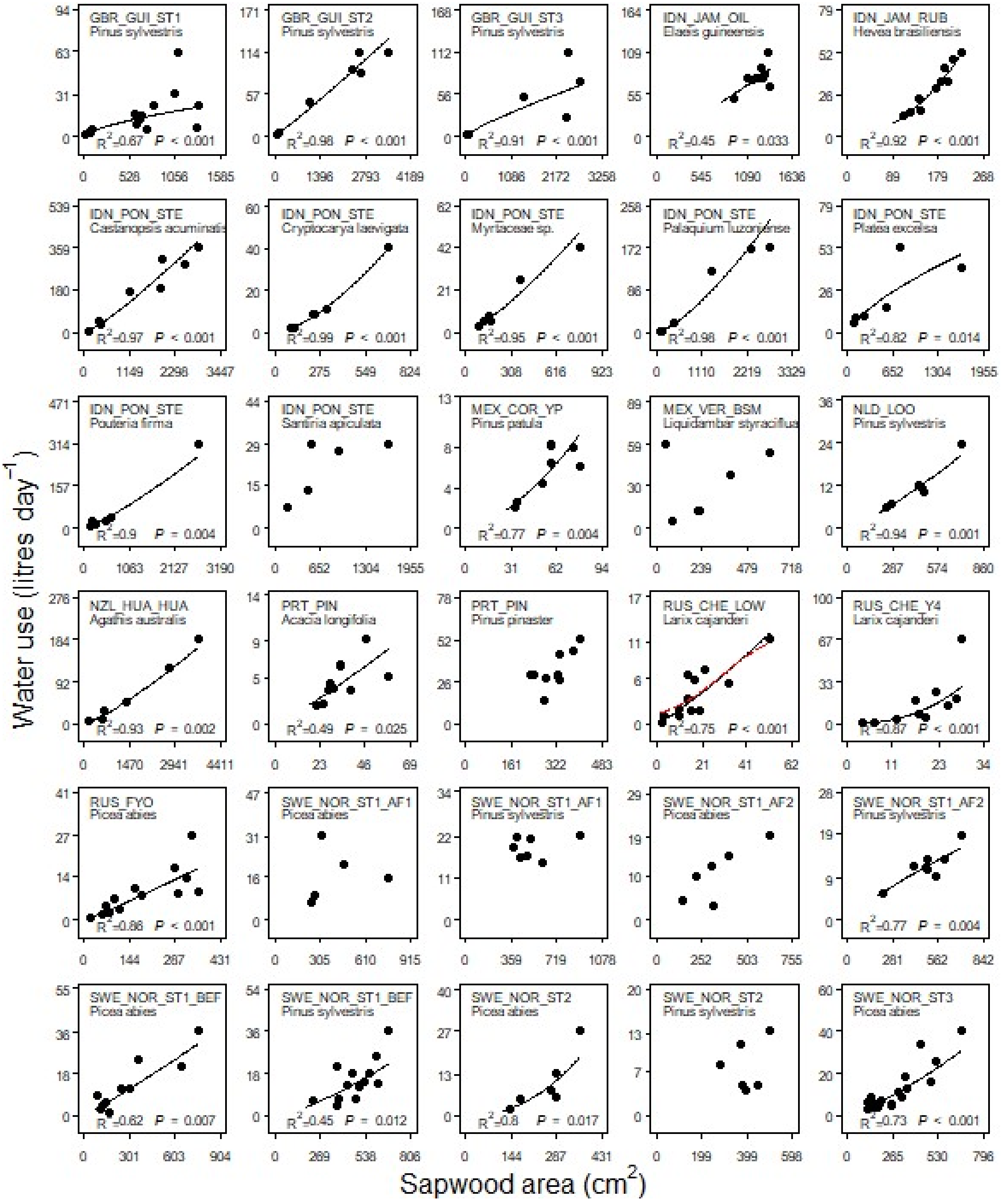

Figure S13. Sapwood area – water use relationships for individual species (figure 4 of 6). When significant, power functions are shown as black lines and sigmoidal functions are shown as red lines.

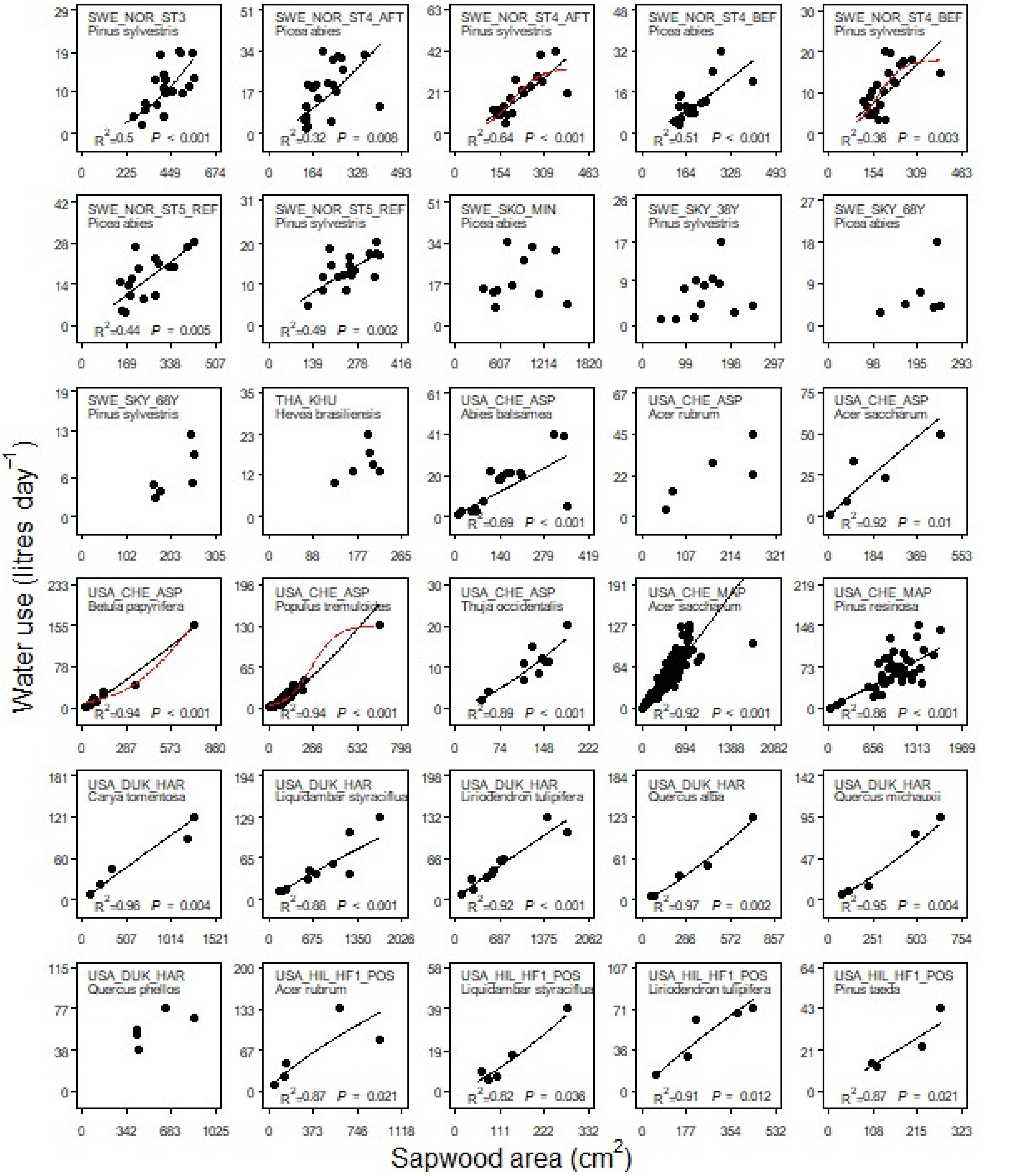

Figure S14. Sapwood area – water use relationships for individual species (figure 5 of 6). When significant, power functions are shown as black lines and sigmoidal functions are shown as red lines.

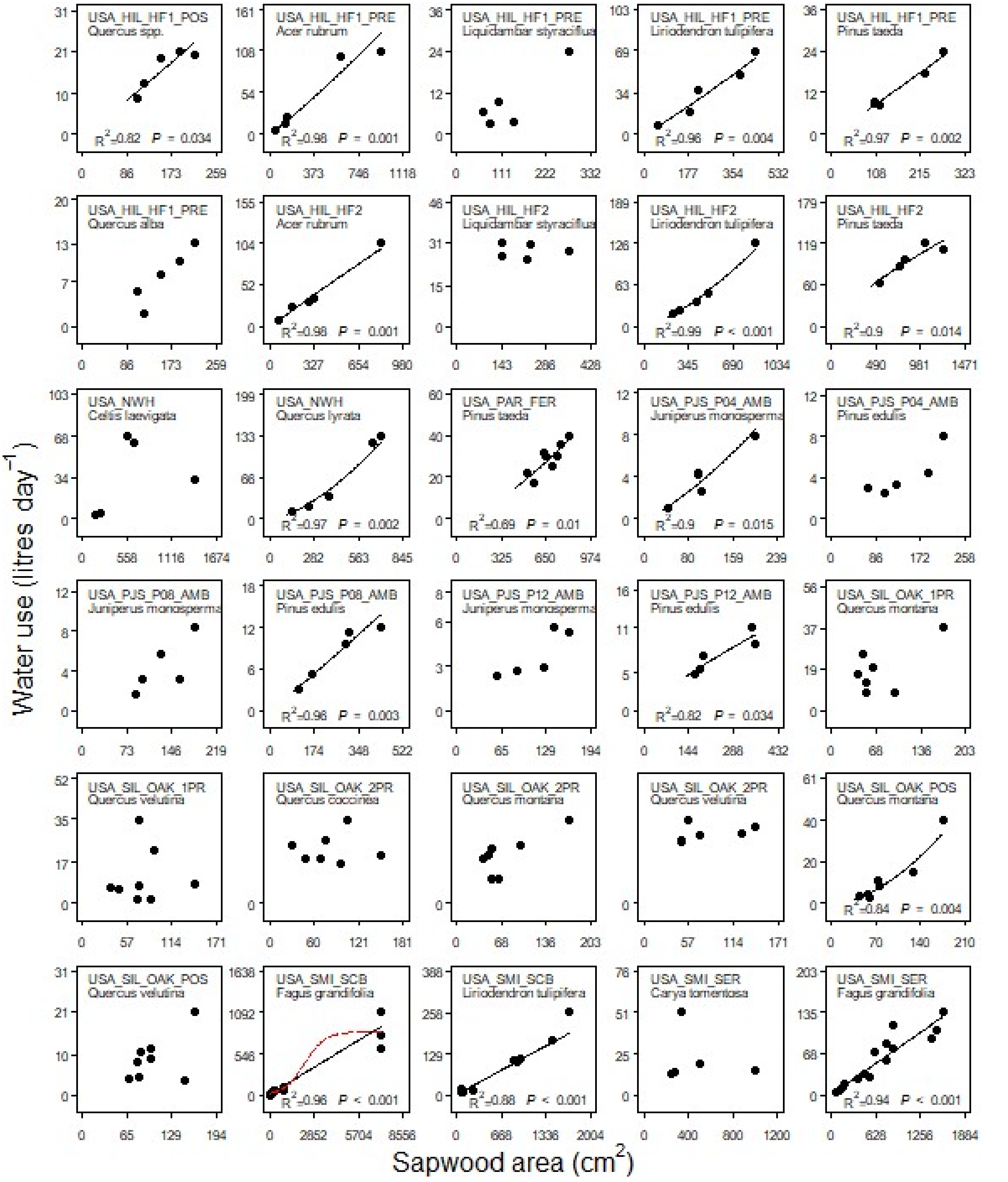

Figure S15. Sapwood area – water use relationships for individual species (figure 6 of 6). When significant, power functions are shown as black lines and sigmoidal functions are shown as red lines.

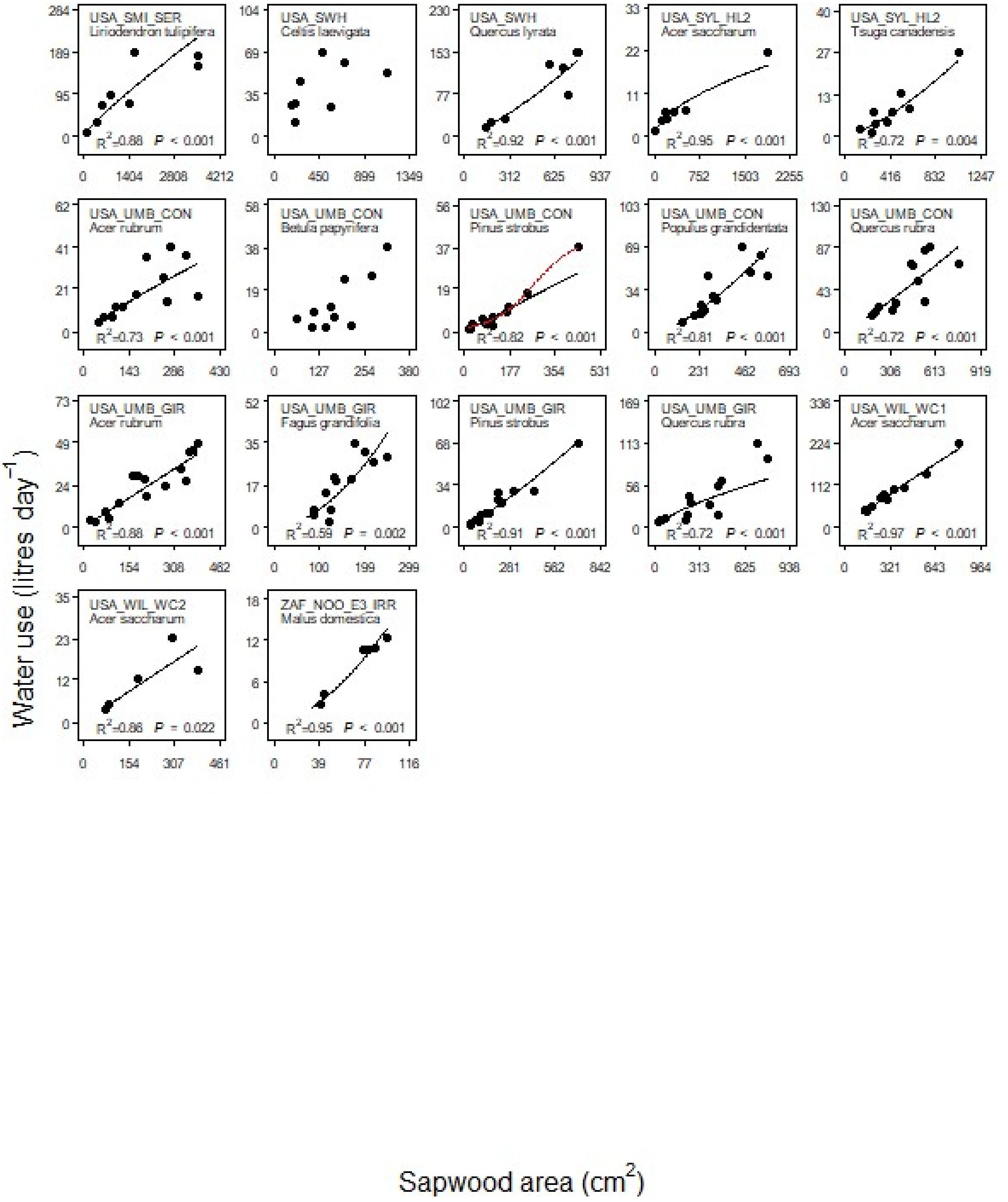

Figure S16. Diameter – sap flux density relationships for individual species (figure 1 of 6).

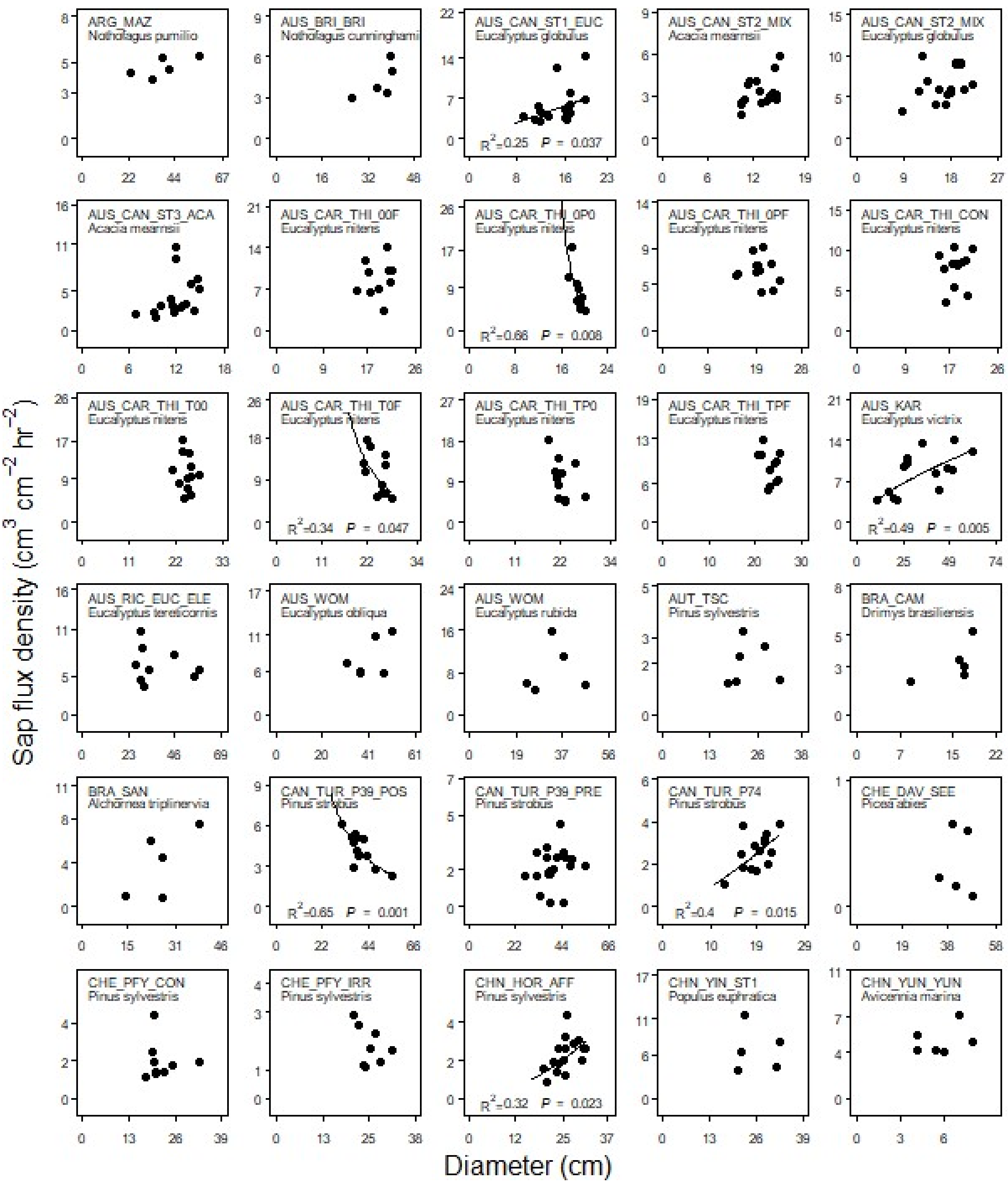

Figure S17. Diameter – sap flux density relationships for individual species (figure 2 of 6).

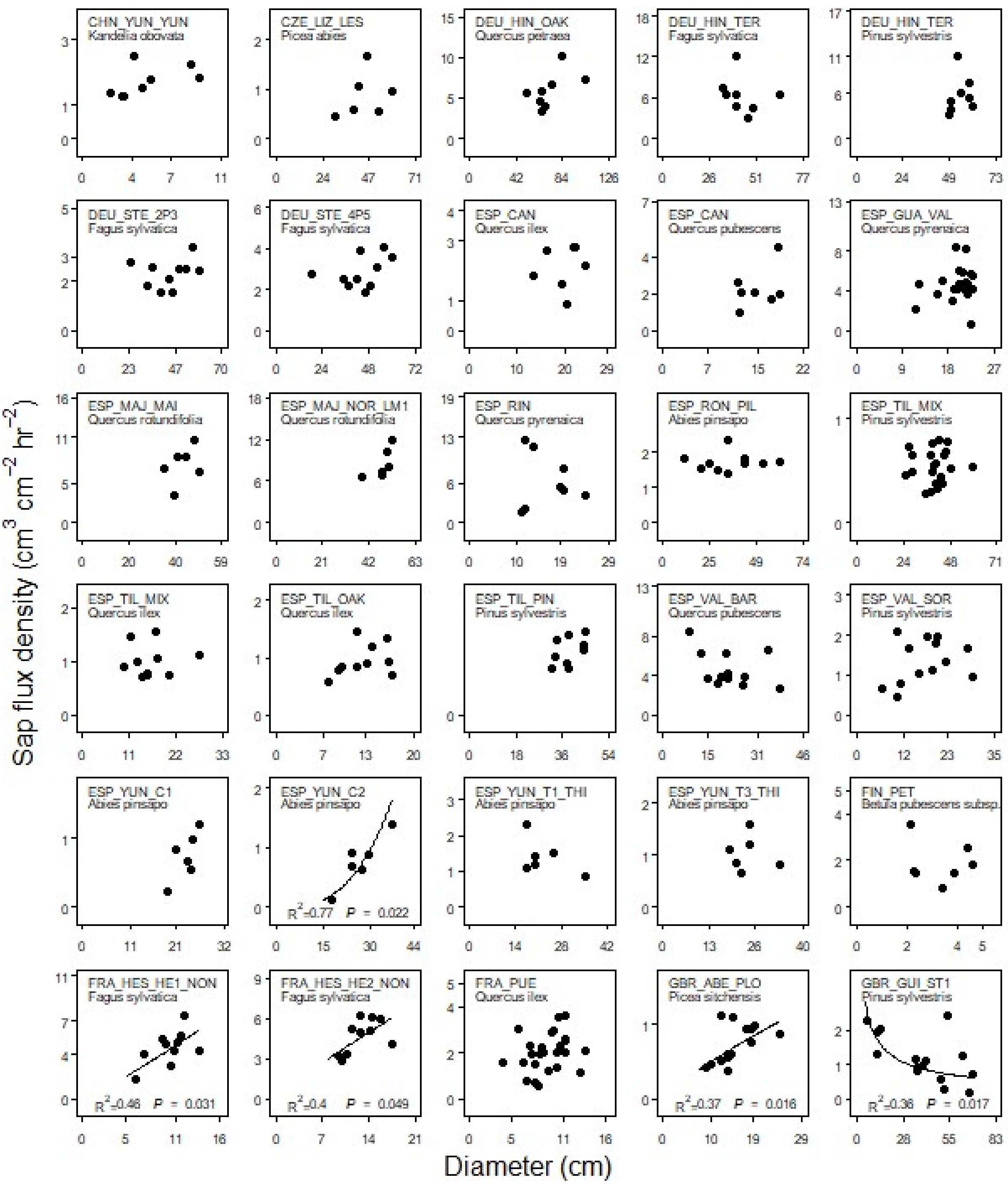

Figure S18. Diameter – sap flux density relationships for individual species (figure 3 of 6).

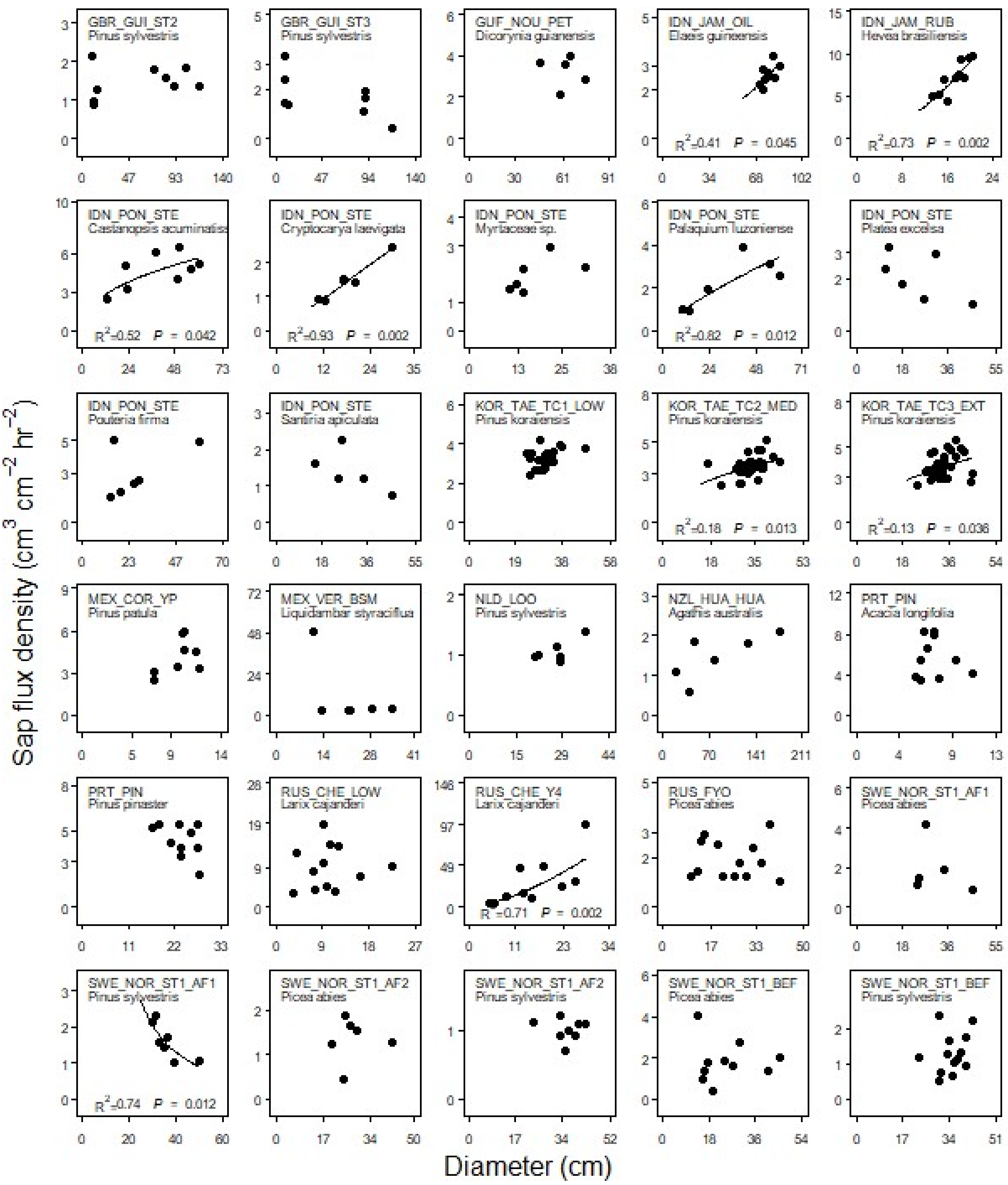

Figure S19. Diameter – sap flux density relationships for individual species (figure 4 of 6).

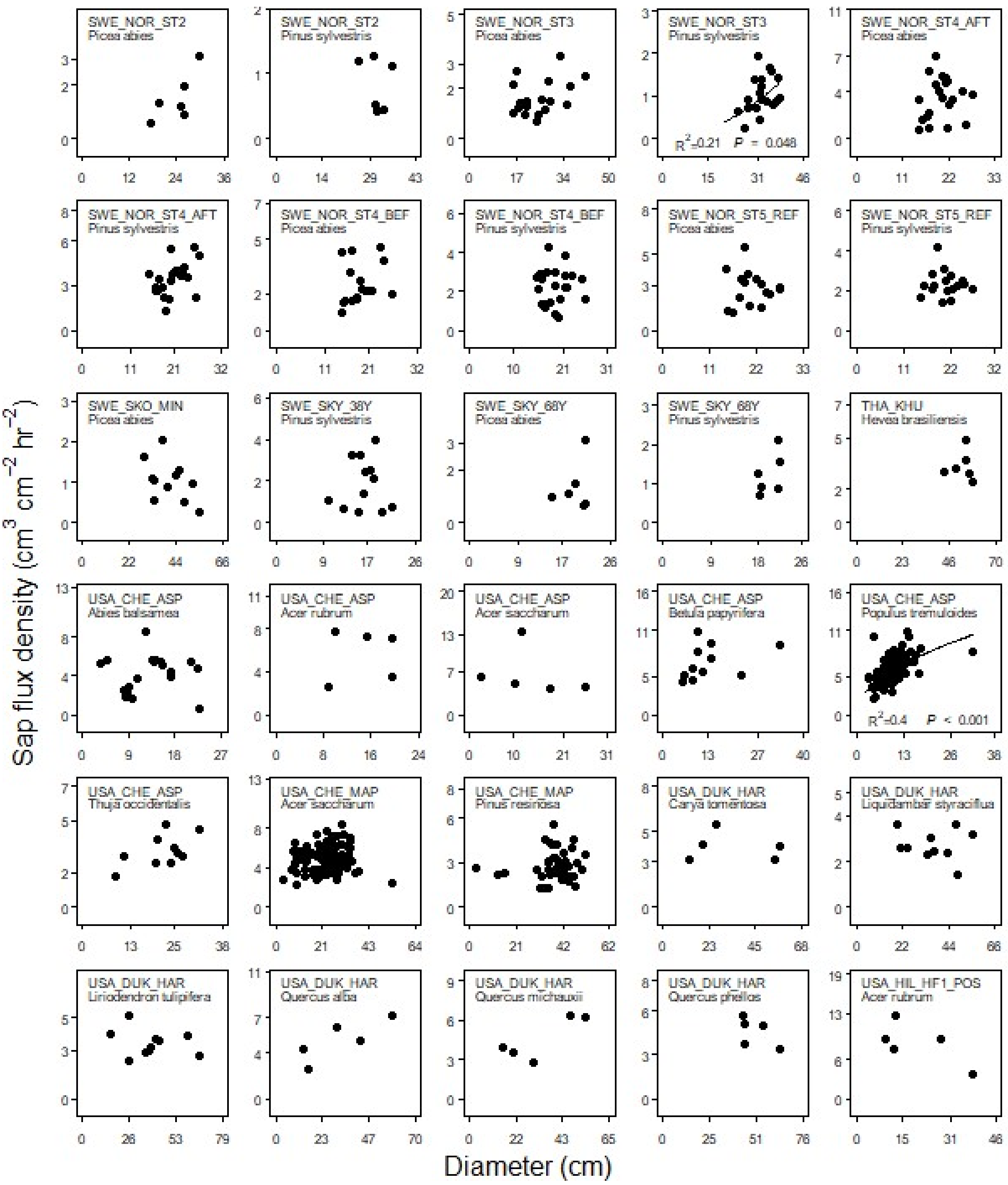

Figure S20. Diameter – sap flux density relationships for individual species (figure 5 of 6).

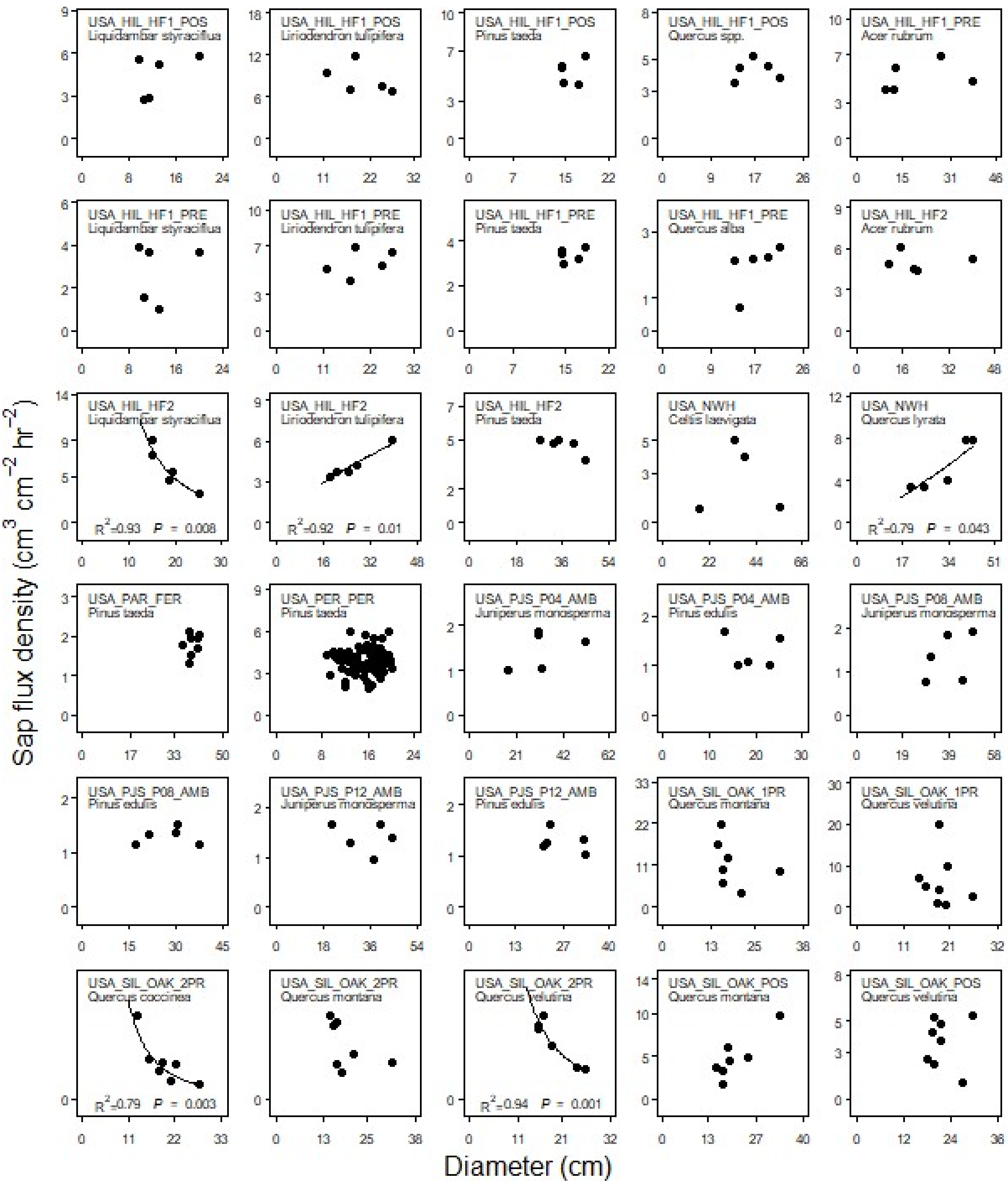

Figure S21. Diameter – sap flux density relationships for individual species (figure 6 of 6).

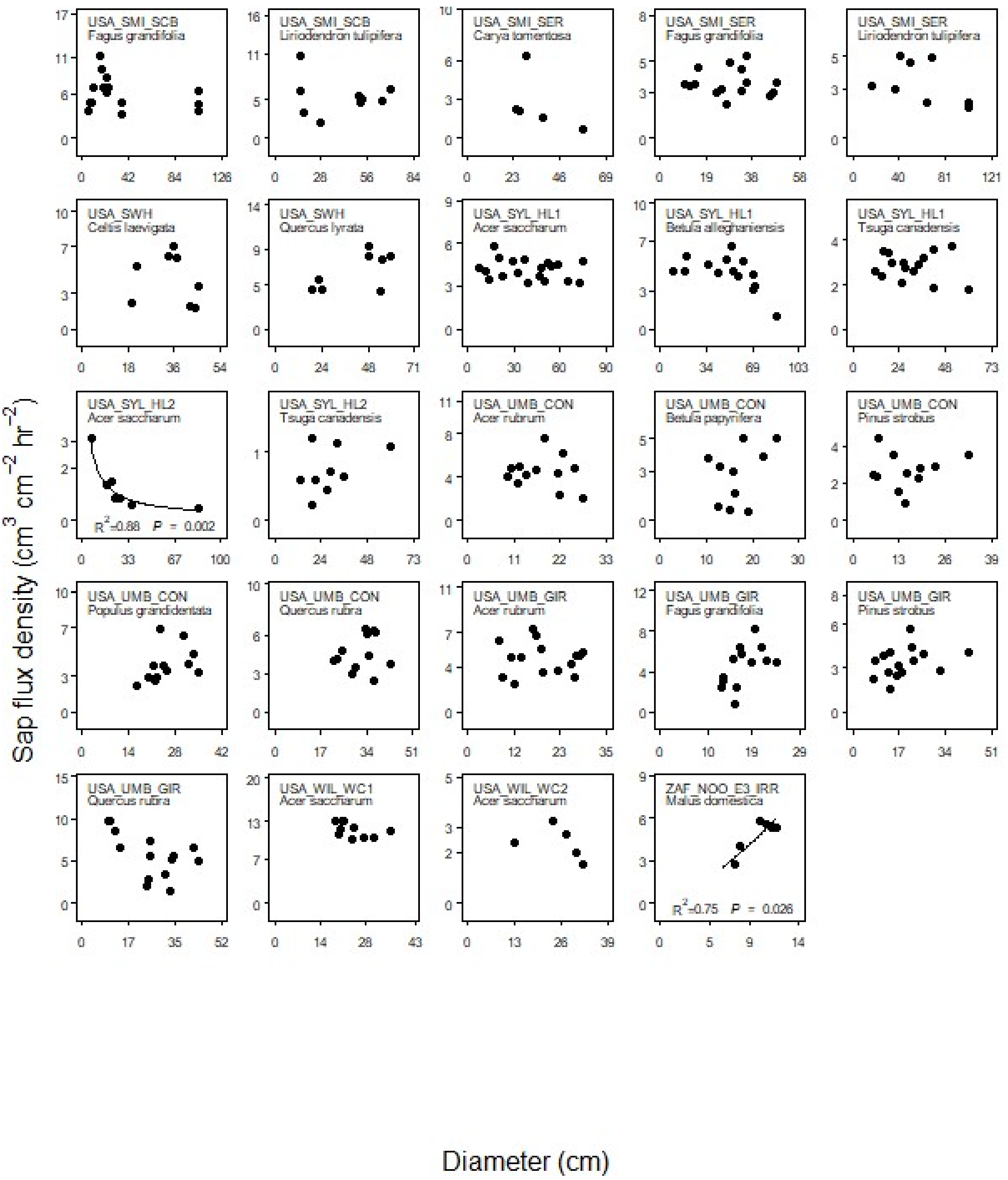

Figure S22. Sapwood area – sap flux density relationships for individual species (figure 1 of 6).

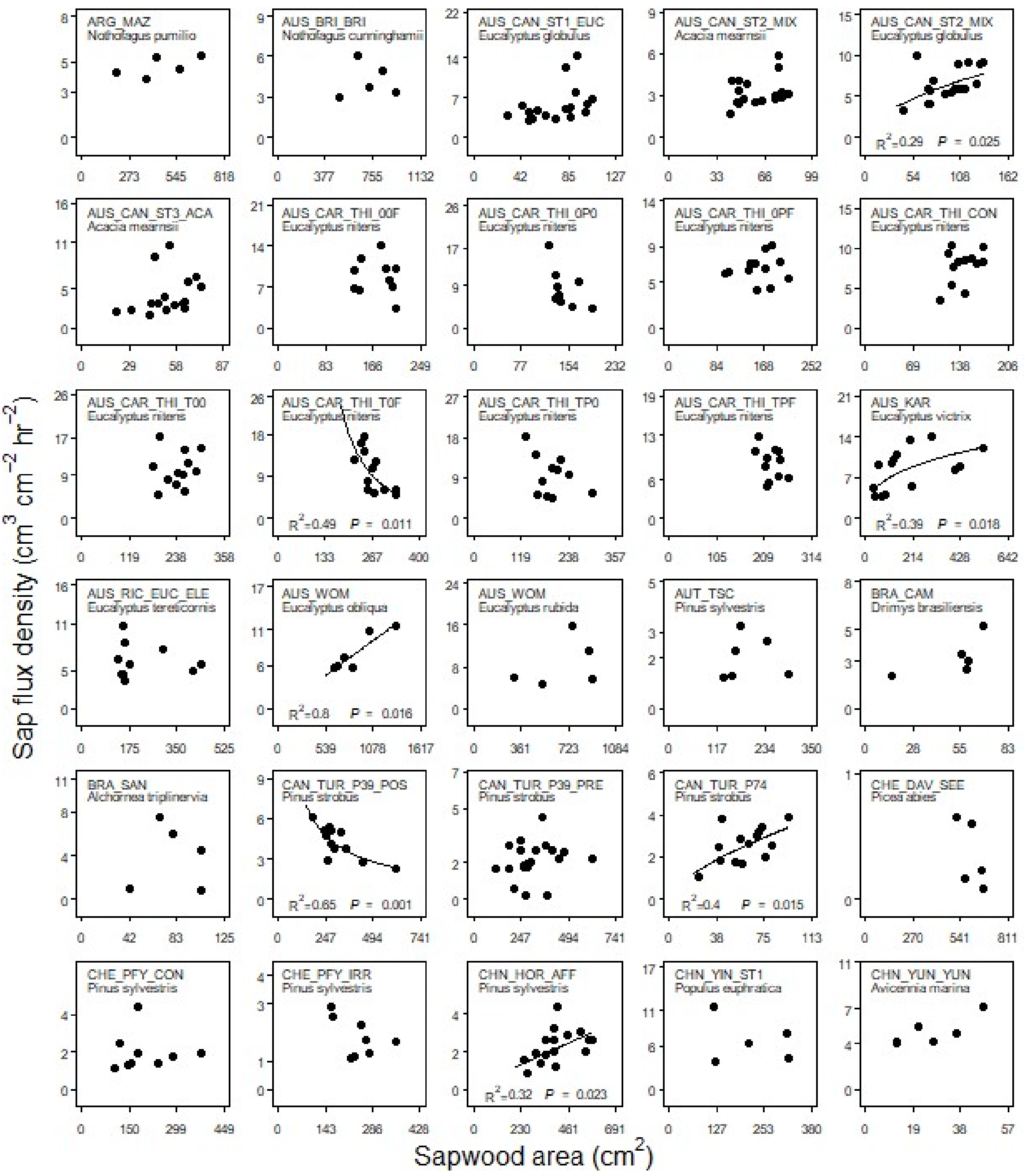

Figure S23. Sapwood area – sap flux density relationships for individual species (figure 2 of 6).

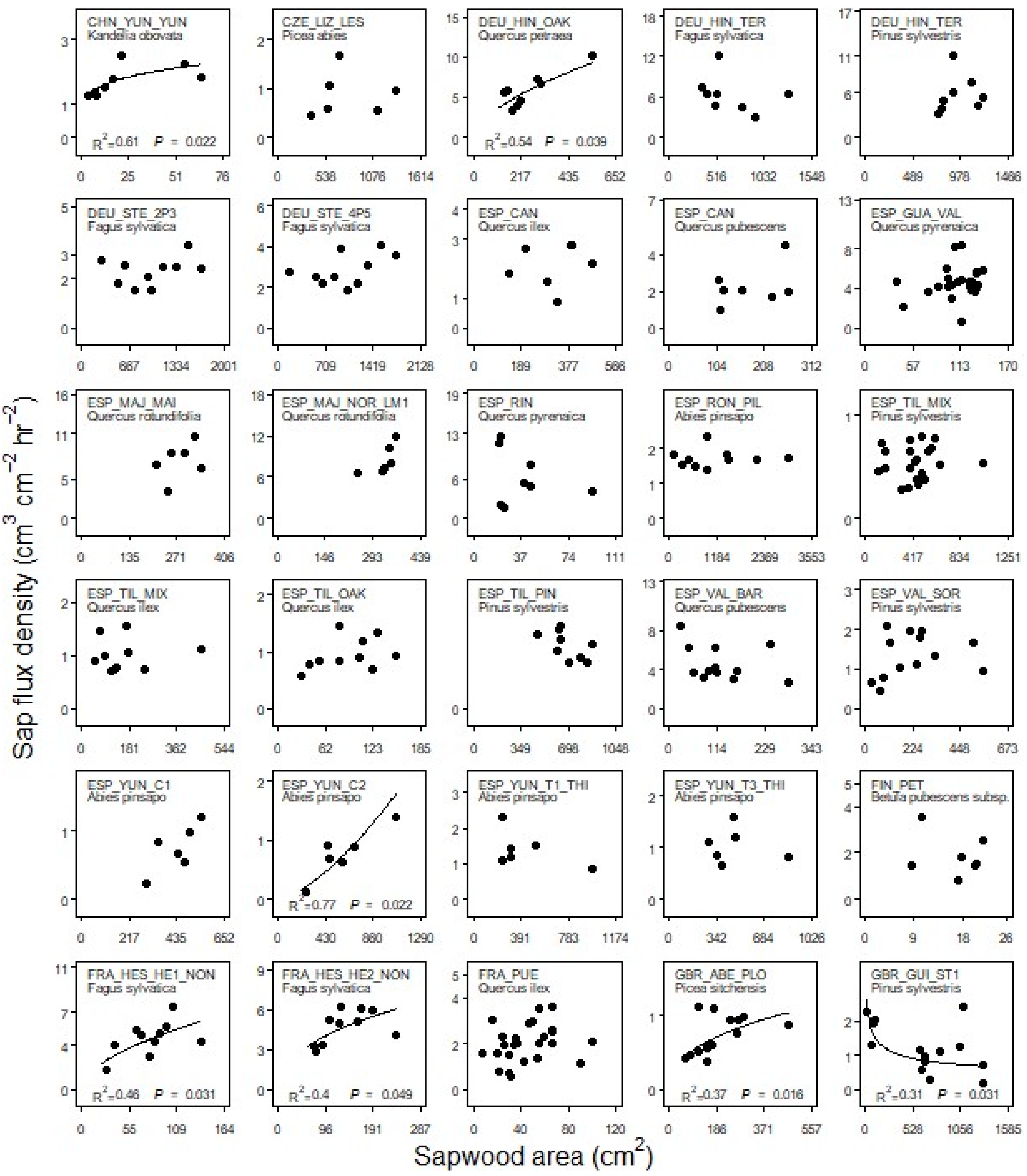

Figure S24. Sapwood area – sap flux density relationships for individual species (figure 3 of 6).

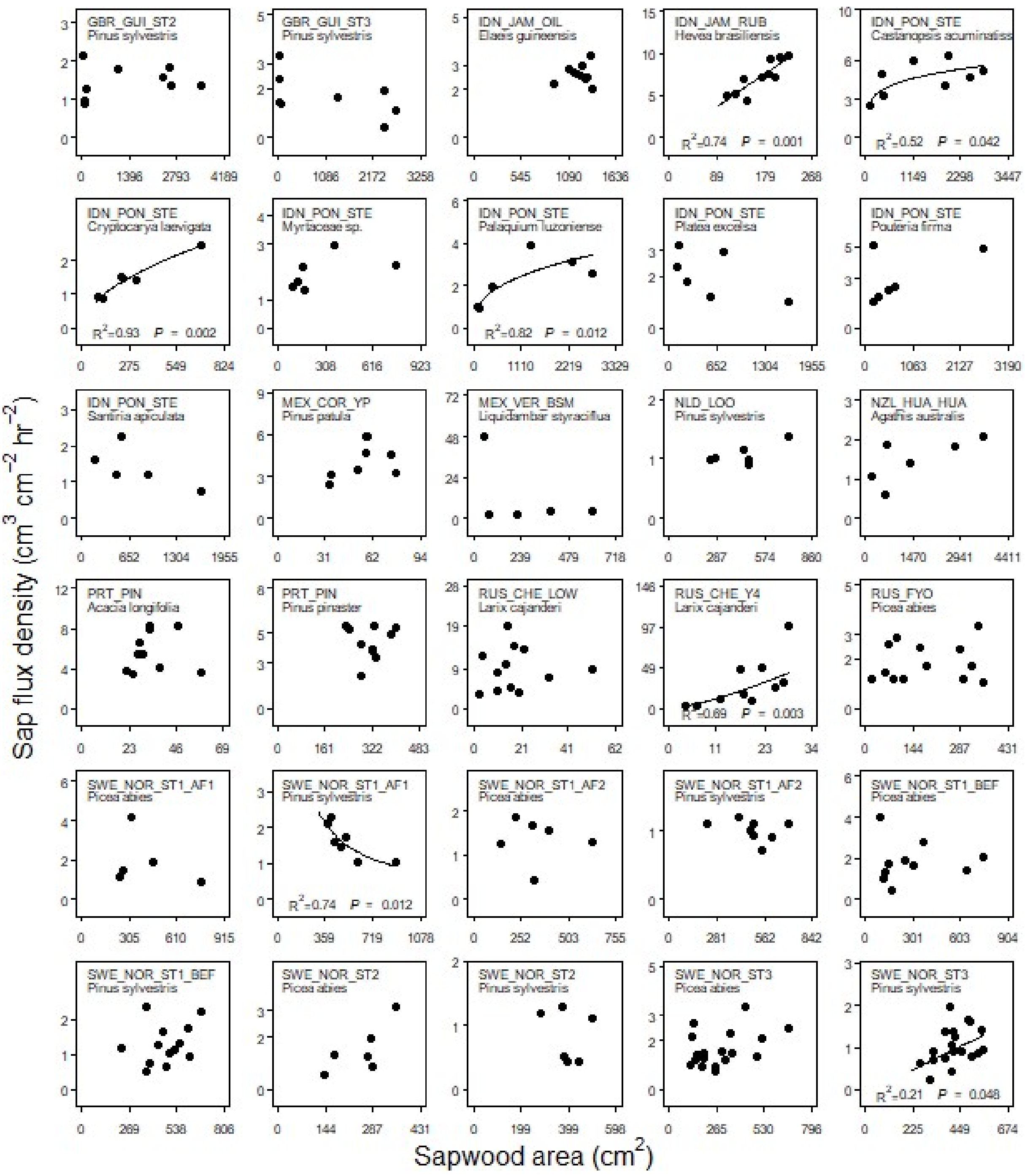

Figure S25. Sapwood area – sap flux density relationships for individual species (figure 4 of 6).

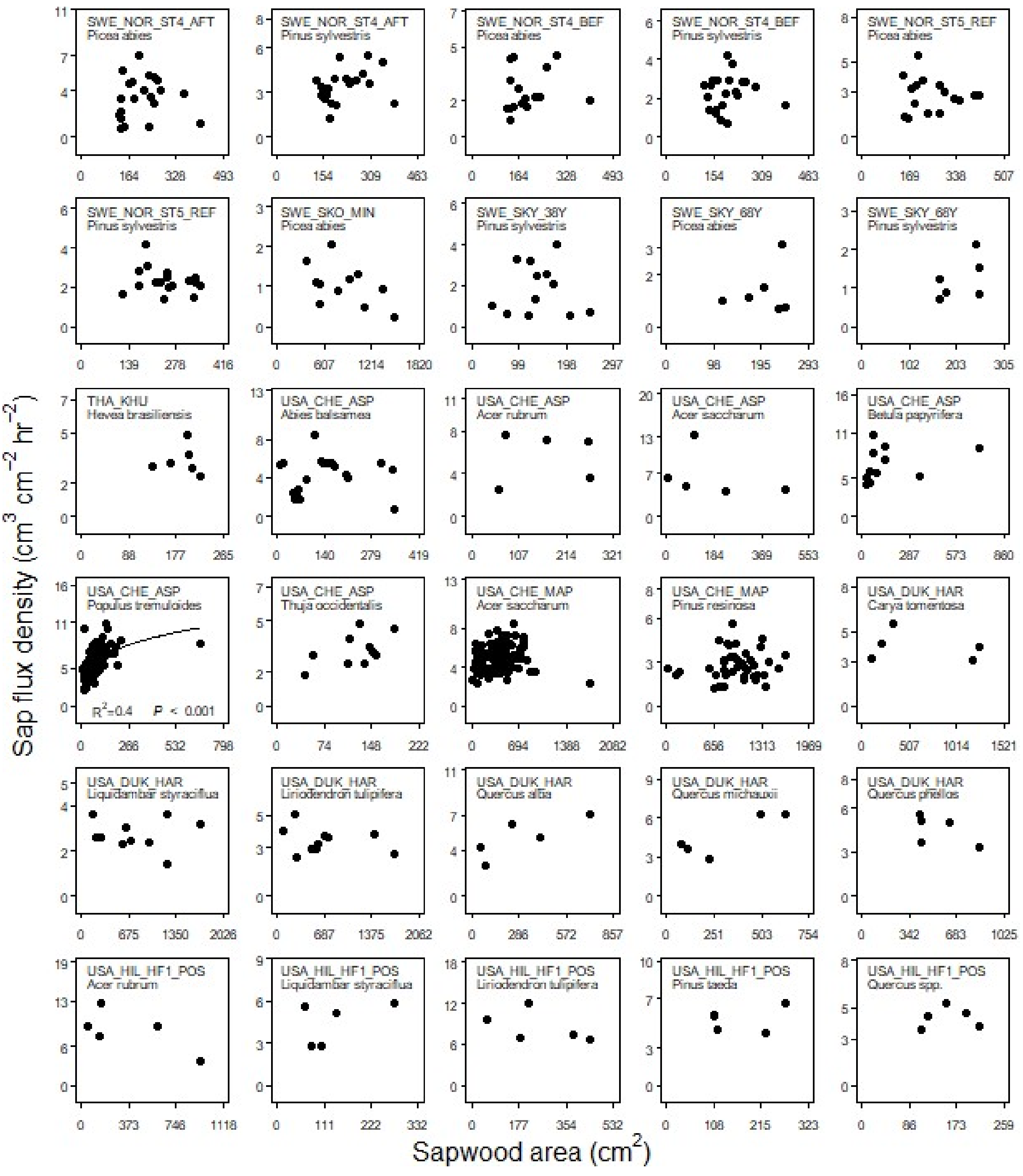

Figure S26. Sapwood area – sap flux density relationships for individual species (figure 5 of 6).

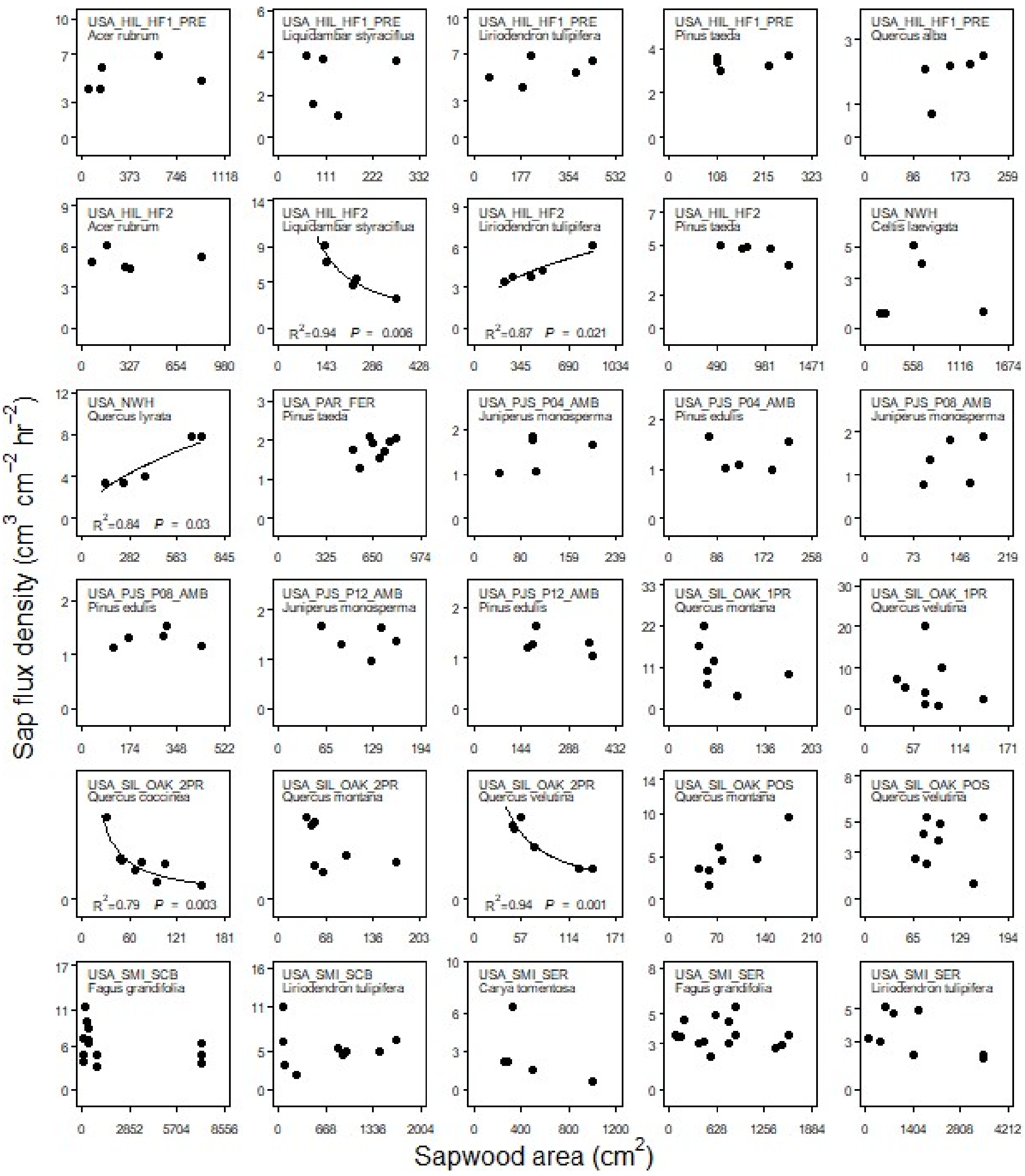

Figure S27. Sapwood area – sap flux density relationships for individual species (figure 6 of 6).

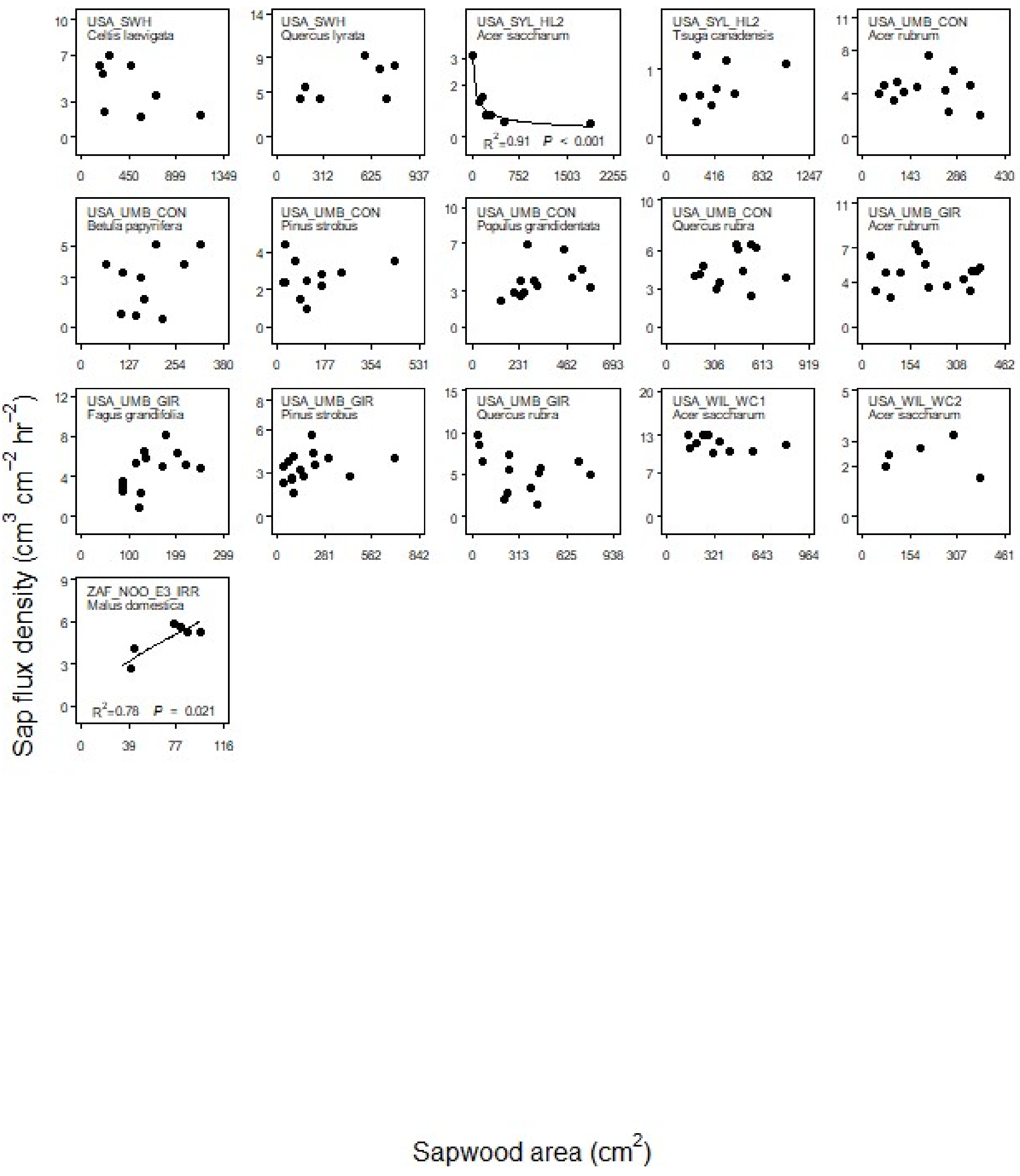

References

Poyatos, R., Granda, V., Flo, V., Adams, M.A., Adorján, B., Aguadé, D., Aidar, M.P.M., Allen, S., Alvarado-Barrientos, M.S., Anderson-Teixeira, K.J., Aparecido, L.M., Arain, M.A., Aranda, I., Asbjornsen, H., Baxter, R., Beamesderfer, E., Berry, Z.C., Berveiller, D., Blakely, B., Boggs, J., Bohrer, G., Bolstad, P.V., Bonal, D., Bracho, R., Brito, P., Brodeur, J., Casanoves, F., Chave, J., Chen, H., Cisneros, C., Clark, K., Cremonese, E., David, J.S., David, T.S., Delpierre, N., Desai, A.R., Do, F.C., Dohnal, M., Domec, J.-C., Dzikiti, S., Edgar, C., Eichstaedt, R., El-Madany, T.S., Elbers, J., Eller, C.B., Euskirchen, E.S., Ewers, B., Fonti, P., Forner, A., Forrester, D.I., Freitas, H.C., Galvagno, M., Garcia-Tejera, O., Ghimire, C.P., Gimeno, T.E., Grace, J., Granier, A., Griebel, A., Guangyu, Y., Gush, M.B., Hanson, P., Hasselquist, N.J., Heinrich, I., Hernandez-Santana, V., Herrmann, V., Hölttä, T., Holwerda, F., Hongzhong, D., Irvine, J., Ayutthaya, S.I.N., Jarvis, P.G., Jochheim, H., Joly, C.A., Kaplick, J., Kim, H.S., Klemedtsson, L., Kropp, H., Lagergren, F., Lane, P., Lang, P., Lapenas, A., Lechuga, V., Lee, M., Leuschner, C., Limousin, J.-M., Linares, J.C., Linderson, M.-L., Lindroth, A., Llorens, P., López-Bernal, Á., Loranty, M.M., Lüttschwager, D., Macinnis-Ng, C., Maréchaux, I., Martin, T.A., Matheny, A., McDowell, N., McMahon, S., Meir, P., Mészáros, I., Migliavacca, M., Mitchell, P., Mölder, M., Montagnani, L., Moore, G.W., Nakada, R., Niu, F., Nolan, R.H., Norby, R., Novick, K., Oberhuber, W., Obojes, N., Oishi, C.A., Oliveira, R.S., Oren, R., Ourcival, J.-M., Paljakka, T., Perez-Priego, O., Peri, P.L., Peters, R.L., Pfautsch, S., Pockman, W.T., Preisler, Y., Rascher, K., Robinson, G., Rocha, H., Rocheteau, A., Röll, A., Rosado, B., Rowland, L., Rubtsov, A.V., Sabaté, S., Salmon, Y., Salomón, R.L., Sánchez-Costa, E., Schäfer, K.V.R., Schuldt, B., Shashkin, A., Stahl, C., Stojanović, M., Suárez, J.C., Sun, G., Szatniewska, J., Tatarinov, F., Tesař, M., Thomas, F.M., Tor-ngern, P., Urban, J., Valladares, F., Tol, C.v.d., Meerveld, I.v., Varlagin, A., Voigt, H., Warren, J., Werner, C., Werner, W., Wieser, G., Wingate, L., Wullschleger, S., Yi, K., Zweifel, R., Steppe, K., Mencuccini, M., Martínez-Vilalta, J., 2021. Global transpiration data from sap flow measurements: the SAPFLUXNET database. Earth System Science Data 13, 2607-2649.
